# Variations in bone mineral density after joint replacement: A systematic review examining different anatomical regions, fixation techniques and implant design

**DOI:** 10.1002/jeo2.70187

**Published:** 2025-05-20

**Authors:** Domenico Alesi, Raffaele Zinno, Maria Scoppolini Massini, Giuseppe Barone, Davide Valente, Erika Pinelli, Stefano Zaffagnini, Agostino Igor Mirulla, Laura Bragonzoni

**Affiliations:** ^1^ Department of Biomedical and Neuromotor Sciences (DIBINEM) University of Bologna Bologna Italy; ^2^ 2nd Orthopaedic and Traumatologic Clinic, IRCCS Istituto Ortopedico Rizzoli Bologna Italy; ^3^ Department for Life Quality Studies (QUVI) University of Bologna Rimini Italy; ^4^ Department of Engineering University of Palermo Palermo Italy

**Keywords:** bone mineral density, fixation technique, implant design, regions of interest, total hip replacement, total knee replacement

## Abstract

**Purpose:**

This study aims to evaluate postoperative periprosthetic bone mineral density (BMD) at various time points following joint replacement with different implant designs and fixation techniques.

**Methods:**

Database search was conducted on MEDLINE, Scopus, Cochrane Central Register of Controlled Trials, Web of Science, and CINAHL for studies analyzing bone remodelling after joint replacement (March 2002–January 2024). Inclusion criteria: English‐language articles; total joint replacement; at least two BMD evaluations; observational studies, cross‐sectional, prospective, retrospective, randomised controlled trials, and clinical trials. Exclusion criteria: no BMD measurement within one month after surgery; BMD data only expressed as percentage changes or graphs without numerical values; no Gruen zone evaluation for hip replacement; no periprosthetic bone evaluation for knee replacement; pharmacological treatment or comorbidities affecting BMD; revision joint replacements; irrelevant articles; no full text or no original data.

**Results:**

Sixty‐eight articles matched the selection criteria. Fifty‐five focused on the hip joint, 12 on the knee, and one on the shoulder. After total hip arthroplasty, the greatest bone resorption occurred in the proximal femur, peaking at 6 months. Cemented implants and tapered stems showed greater bone resorption than cementless implants and anatomical stems. BMD around the acetabular component decreased during the first 6 months but increased in regions subjected to higher loads. In total knee arthroplasty, bone loss occurred in the anterior distal femur and medial tibial plateau, with cemented and posterior‐stabilised implants showing greater bone loss than cementless and cruciate‐retaining designs.

**Conclusions:**

The periprosthetic BMD decreases progressively after joint replacement. The fixation technique and implant design influence the extent and pattern of this decline. These factors must be considered during the surgical planning, as they can have long‐term implications for bone health and implant longevity. Further research is needed to optimise implant design and surgical techniques to mitigate BMD loss and improve patient outcomes.

**Level of Evidence:**

Level IV.

**Abbreviations**
AMK: anatomic Modular Knee
AML: anatomic medullary locking
BFH: big femoral head
BMD: bone mineral density
BMI: body mass index
CR: cruciate retaining
d: day
DXA: dual x‐rays absorptiometry
HA: hydroxyapatite
JBI: Joanna Briggs Institute
m: month
PCL: Posterior cruciate ligament
PPS: standard porous coated
PRISMA: Reporting Items for Systematic Reviews and Meta‐analyses
PS: posterior stabilised
RHA: resurfacing hip arthroplasty
ROB2: version 2 of the Cochrane risk‐of‐bias tool for randomised trials
ROI: regions of interest
SPECT: single photon emission computed tomography
Surg.: surgery
THA: total hip arthroplasty
TKA: total knee arthroplasty
TMT: trabecular metal technology
TOP: trabeculae‐oriented pattern
UKA: unicompartmental knee arthroplasty
w: week

## INTRODUCTION

Periprosthetic bone remodelling represents a topic of great interest in the orthopaedic community due to its implications on implant survival. Indeed, a decrease in bone mineral density (BMD) around the implant is linked to a higher risk of complications such as fractures and loosening [84]. Although designs and materials have evolved, loosening is still one of the leading causes for implant failure [44, 89]. This process seems to be induced by stress shielding, or the variation of BMD in the periprosthetic bone in response to the different load forces distribution caused by the implant rigidity [70, 95].

The current gold standard to analyze BMD changes around an orthopaedic implant is the dual‐energy x‐rays absorptiometry (DXA), which is able to provide accurate and reproducible measurements with minimal radiation exposure [8, 19, 45]. Studies utilising DXA have predominantly focused on total hip arthroplasty (THA), reflecting the high global incidence of hip replacements and the structured research methodologies available for this joint [102]. To allow a fair comparison between the results obtained by various authors, Gruen et al. introduced a standardised subdivision of the regions of interest (ROI) around the femoral stem [35]. Using this method, various studies have shown that multiple variables like body mass index (BMI), sex, comorbidities, pharmacological treatments, implant design and fixation technique can affect periprosthetic bone health, functional recovery, adverse events and revision rate [5, 57, 69, 71, 87, 98], but the information obtained from that amount of data is very heterogeneous.

On the contrary, studies analyzing BMD changes after total knee arthroplasty (TKA) are rather limited and there is no standardised subdivision of periprosthetic ROIs among the various authors allowing a systematic analysis. Furthermore, studies that have focused on BMD changes after other joint replacements are isolated and performed on small samples.

Given the fragmented nature of the existing literature, a comprehensive review which collects data from multiple studies is needed, to provide easy access to information on BMD changes based on specific subcategories of the patient population and help to overcome the limitations of the current designs. Hence, the aim of this systematic review was to provide an overview on the changes of periprosthetic BMD after joint replacement considering different implant designs and fixation technique.

## METHODS

The systematic review was conducted in accordance with the Reporting Items for Systematic Reviews and Meta‐analyses (PRISMA) guidelines [72, 79]. The systematic review's protocol was registered in the International Prospective Register of Systematic Reviews (CRD42023401291).

### Eligibility criteria

PICOS (Patients, Interventions, Comparators, Outcomes and Study design) question was developed using the following search terms: (P) People aged 18 or more; (I) Total joint prosthesis surgery; (C) early prosthesis surgery; (O) Bone mineral density; (S) Observational studies, cross‐sectional, prospective, retrospective, randomised controlled trial and clinical trials. Studies available in full text, published in English, with original primary data, and published after 2002 were included. There was no limitation for gender or type of prostheses.

The inclusion criteria were the following: (i) articles written in English; (ii) patients who underwent total joint replacement; (iii) at least two BMD evaluations per patient; (iv) observational studies, cross‐sectional, prospective, retrospective, randomised controlled trial and clinical trials. The exclusion criteria were the following: (i) no BMD measurement within one month after surgery; (ii) BMD expressed as only percentual changes or graphs, without numerical values; (iii) no Gruen zone (ROI) evaluation for hip replacement; (iv) no periprosthetic bone evaluation for knee replacement; (v) pharmacological treatment (such as steroids, bisphosphonates, estrogens etc…) or comorbidities that could affect the BMD; (vi) revision joint replacements; (vii) articles not relevant for the research area and (viii) no full text available or no original data.

### Search strategy and data sources

The literature search was performed by searching the following databases: MEDLINE (PubMed), Scopus, Cochrane Central Register of Controlled Trials, Web Of Science, and CINAHL. The databases were consulted on January 16th, 2024. Search strategy was created following the search string, with terms and Boolean logical operator, used on the PubMed. The keywords used for the screening were related to bone mineral density and joint arthroplasty. The strings were adapted to meet the specific search requirements of each database. The complete strings for each database are available in the supplementary material (Supporting Information: Annex A ‐ Table S1). Moreover, a grey literature search of other papers was conducted using hand searches of key conference proceedings, journals, professional organisations' websites and guideline clearing houses. Finally, the snowball technique was used to examine references cited in the primary papers to identify potential papers that fit the eligibility criteria and could be included in this review. Among the complete list of items found for each database, duplicate articles were excluded using EndNote (EndNote X9.3.3) and then a manual verification was conducted.

Based on the PICOS criteria, the titles and abstracts were screened by eight authors (D.A., R.Z., M.S.M., G.B., D.V., E.P., A.I.M. and L.B), and studies that did not meet the purpose of the present review were excluded. Then, full texts of all remaining papers were reviewed to identify which could be included in this article. Moreover, each author individually screened all studies. Title, abstract and full texts were checked twice to minimise the risk of missing relevant articles. Any uncertainties or disagreements regarding inclusion or exclusion were discussed by all authors together. Five authors (D.A., R.Z., A.I.M., G.B. and D.V.) extracted data from included studies following a formatted table to standardise data collection rules. The data collected includes first author's name, journal name (quartile and year of publication), study design, aim, population, joint, materials and methods, assessment time (follow up), type of prosthesis, type of implant (cemented or cementless) and outcomes. The study's authors were contacted to have additional information where necessary.

The variable analysed in the present review was BMD. The weighted average of BMD values (g/cm^2^) postoperatively (baseline) and at subsequent follow‐ups was calculated. Because variability was present in the baseline BMD values of the included studies, the variation between baseline BMD and subsequent follow‐ups was calculated as percentage. The data analysis was performed using Microsoft® Excel (version 2402).

### Study selection

**Figure 1:**
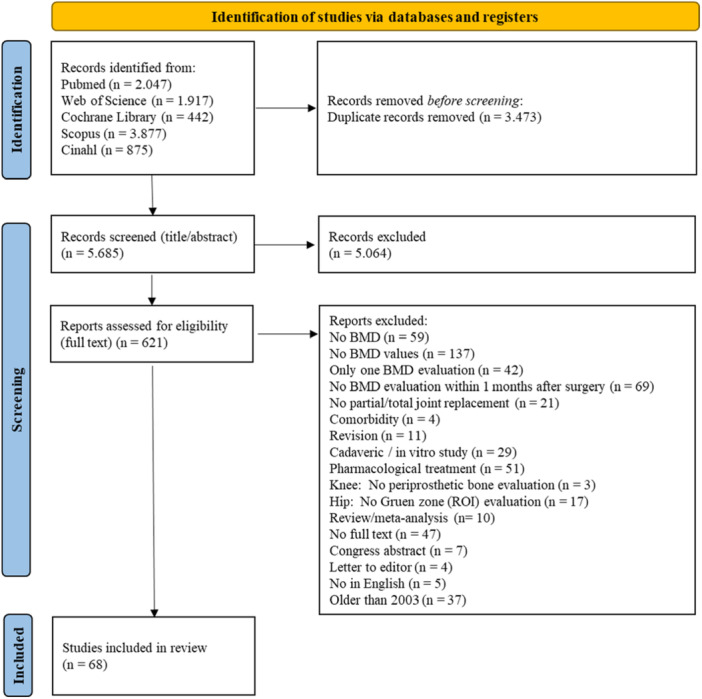
PRISMA flow diagram.

A total of 9158 records were identified through database screening (PUBMED: 2047; WEB OF SCIENCE: 1917; COCHRANE LIBRARY: 442; SCOPUS: 3877; CINAHL: 875), of which 3473 were removed as duplicates by EndNote. Then, after screening titles and abstracts, 5064 more were excluded (774 more duplicates and 53 study protocols). Finally, after full text screening according to the exclusion criteria, 68 out of 621 articles were included in the systematic review (Figure 1). Descriptive statistics were used to summarise and present the results.

### Quality assessment and risk of bias

A Risk of bias critical appraisal of each article included in the review was conducted independently and blinded by three authors (EP, MSM and LB), using the “Revised Cochrane risk‐of‐bias tool for randomised trials” (ROB2) for randomised controlled trial [93], and the Joanna Briggs Institute (JBI) Critical Appraisal tools were used according to the specific study design [73]. Any disagreement or conflict between the quality scores separately assigned by the three blind reviewers was discussed and resolved by majority vote. The ROB2 is organised into five bias domains, focusing on various aspects of trial design, conduct and reporting.

The Joanna Briggs Institute Critical Appraisal (JBI) Critical Appraisal tools contain from eight to eleven questions whose answers could be 'yes', 'no', 'unclear', and 'not applicable'. The number of questions depends on the type of study design.

## RESULTS

### Study characteristics

All data necessary for the analysis have been extracted and are presented in Table 1. A great heterogeneity among the studies emerged as the methods of BMD analysis were not standardised among the various authors and among the various joints. Therefore, the articles were grouped according to the evaluated joint, the regions of interest (ROI) adopted, the fixation technique and the implant design, analyzing the percentage change in BMD compared to baseline. The main findings of the subgroups in which a systematic analysis was not possible were presented separately.

**Table 1:** Data extraction.

First Author	Journal name, quartile, year	Study Design	Aim	Population	Joint	Materials and Methods	Assessment time	Type of prosthesis	Type of implant	Outcomes
Ahrens, P [1].	Hip International, Q2 (2004)	Prospective study	To correlate the gross radiographic changes with the DXA	*n* = 19 ‐Mean age = 54 years ‐Age range = 43–62 years Male = 8 Females = 11	Hip	DXA	T0 = Post surg T1 = 32 m T2 = 89 m	Anatomic medullary locking (AML) uncemented femoral component (DePuy, International, Leeds, UK)	Cementless	Gruen Zone ROI 7 Femur
Aldinger, P. R [2].	Calcified Tissue International, Q1 (2003)	Prospective longitudinal study Cross‐sectional study	To evaluate the pattern of periprosthetic bone remodelling around stable uncemented tapered hip stems	*n* = 35 Male = 17 ‐Mean age = 54.6 ± 9.1 years Female = 18 ‐Mean age = 55.2 ± 9.9 years	Hip	DXA	T0 = Post surg T1 = 3 m T2 = 6 m T3 = 12 m T4 = 36 m T5 = 60 m T6 = 84 m	Press‐fit titanium Spotorno stem, Sulzer Orthopaedic	Cementless	Gruen Zone ROI 7 Femur
Alm, J [3].	Acta Orthopaedica, Q1 (2009)	Prospective study	To investigate the association between early changes in periprosthetic BMD and patient‐related factors	*n* = 39 Female = 39 ‐Mean age = 63 years ‐Age range = 41–79 years	Hip	DXA	T0 = Post surg (7 d) T1 = 3 m T2 = 6 m T3 = 12 m T4 = 24 m	Anatomic Benoist Girard II, ABG II, Stryker	Cemented	Gruen Zone ROI 7 Femur
Andersen, M [4].	Journal of Clinic Densitometry: Assessment & Management of Musculoskeletal Health, Q1 (2018)	Prospective study	To investigate the adaptive bone remodelling of the distal femur after TKA using the uncemented Nexgen CR flex femoral component	*n* = 65 ‐Mean age = 61.0 years Male = 30 Female = 35	Knee	DXA	T0 = Post surg T1 = 3 m T2 = 6 m T3 = 12 m T4 = 24 m	Uncemented Titanium Zimmer Nexgen CR‐Flex Femoral Component (Zimmer Inc, Warsaw, IN)	Cementless	ROI 3 Femur
Bieger, R et al.[6]	Hip International, Q2 (2011)	Prospective study	To evaluate differences in periprosthetic BMD in 25 patients undergoing cementless and in 18 patients undergoing cemented unilateral THA using the Optan stem	*n* = 43 Cementless = 25 ‐ Mean age = 61 years ‐ Age range = 42–67 years Cemented = 18 ‐ Mean age = 74 years ‐ Age range = 63–94 years	Hip	DXA	T0 = 14ds T1 = 3 m T2 = 12 m	Uncemented titanium base alloy with porous coated proximal third + Cemented stem made of CoNCrMo alloy wOptan stem (Zimmer Germany GmbH, Freiburg, Germany)	Cemented and Cementless	Gruen Zone ROI 7 Femur
Boller, S et al. [10]	Archives of Orthopaedic and Trauma Surgery (2018)	Prospective study	To examine potential differences between patients under and over 60 years who underwent a total short hip stem arthroplasty in a 24‐month follow‐up in a clinical setting	*n* = 67 <60 years = 39 ‐Mean age = 50.9 years ‐SD = 6.4 >60 years = 28 ‐Mean age = 66.3 years ‐SD = 5.5	Hip	DXA	T0 = Post surg T1 = 6 m T2 = 12 m T3 = 24 m	Metha® (BBraun, Aesculap, Tuttlingen, Germany) short hip stem prosthesis	Cementless	Gruen Zone ROI 7 Femur
Brinkmann, V et al. [12]	Journal of Orthopaedics and Traumatology, Q2 (2015)	Prospective randomised study	To investigate osseointegration and bone remodelling after implantation of the Metha™ or Nanos™ prostheses, to analyze whether proximal load transfers could be achieved and whether there are differences between the two implants	*n* = 50 ‐Mean age = 58.7 years ‐Age range = 43–70 years Metha™ = 24 ‐ Male = 12 ‐ Female = 12 ‐ Mean age = 58.7 years Nanos™ = 26 ‐Male = 16 ‐Female = 10 ‐ Mean age = 59.7 years	Hip	DXA	T0 = Post surg T1 = 3 m T2 = 12 m	Metha™ (Aesculap AG, Tuttlingen, Germany) + NanosTM (Smith & Nephew GmbH, Marl, Germany)	Cementless	Gruen Zone ROI 7 Femur
Brinkmann, V et al. [13]	Acta Orthopaedica, Q1 (2017)	Prospective randomised study	To analyze bone remodelling around the Nanos® (Smith & Nephew) and Metha® (Aesculap AG) implants as a function of varus/valgus stem positioning	*n* = 75 ‐Mean age = 58.7 years ‐Age range = 43–70 years Metha™ = 24 Nanos™ = 51	Hip	DXA	T0 = Post surg (5 d) T1 = 3 m T2 = 12 m	Metha™ (Aesculap AG, Tuttlingen, Germany) + NanosTM (Smith & Nephew GmbH, Marl, Germany)	Cementless	Gruen Zone ROI 7 Femur
Buckland, A et al. [15]	The Journal of Arthroplasty (2010)	Prospective case series	To assess with DXA the changes in periprosthetic BMD around a triple‐taper stem, with particular attention to the changes in proximal femoral BMD to identify the relationship between age, sex, preoperative BMD, mobility, and surgical approach to postoperative changes in calcar BMD	n = 103 ‐Mean age = 71.6 years ‐Age range = 61–88 years Male = 47 Female = 56	Hip	DXA	T0 = Post surg T1 = 3 m T2 = 9 m T3 = 18 m T4 = 24 m	Highly polished, triple‐taper, cemented C‐stem (DePuy, Warsaw, Ind)	Cemented	Gruen Zone ROI 7 Femur
Burchard, R et al. [17]	Archives of Orthopaedic and Trauma Surgery, Q1 (2007)	Prospective study	To collect prospective medium term (5 years) volumetric CT density data after cemented femoral stem implantation	*n* = 7 ‐Mean age = 63.9 years	Hip	Volumetric CT density	T0 = Post surgery T1 = 24 m T2 = 60 m	Marburg system; Sulzer Orthopedics	Cemented	Gruen Zone ROI 7 Femur
Christiansen, J et al. [18]	The Journal of Bone and Joint Surgery (2020)	Pilot study	To evaluate the 2‐year performance of the Primoris in terms of implant migration and BMD around the implant	*n* = 50 ‐Mean age = 52 years ‐Age range = 25–65 years Male = 45 Female = 5	Hip	DXA	T0 = Post surg (1 d) T1 = 24 m	Primoris femoral neck‐preserving hip implant (Biomet)	Cementless	Gruen Zone ROI 4 Femur
Damborg, F et al. [20]	Acta Orthopaedica, Q1 (2008)	Prospective study	To quantify the changes in BMD for 5 years after insertion of the cemented Exeter stem in women	*n* = 18 Female = 18 ‐Age range: 55–79 years	Hip	DXA	T0 = Post surg T1 = 18 m T2 = 60 m	Exeter stem	Cemented	Gruen Zone ROI 7 Femur
Dan, D et al. [21]	Rheumatology International, Q2 (2006)	Prospective study	To evaluate periprosthetic bone loss and to compare it with the bone loss in other areas of the body	*n* = 50 Male = 40 Female = 10 Cemented = 23 Uncemented = 27	Hip	DXA	T0 = Post surg T1 = 12 m	‐	Cemented and Cementless	Gruen Zone ROI 7 Femur
Decking, R et al. [22]	BMC Musculoskeletal Disorders, Q1 (2008)	Prospective study	To investigate the changes of BMD in the proximal femur and the clinical outcome after implantation of a short femoral‐neck prosthesis	*n* = 20 ‐Mean age = 47 years ‐SD = 11.6 Male = 12 Female = 8	Hip	DXA	T0 = Post surg (10 d) T1 = 3 m T2 = 12 m	ESKA Cut 2000 femoral	Cementless	Gruen Zone ROI 7 Femur
Digas, G et al. [25]	Acta Orthopaedica, Q1 (2006)	Prospective study	To compare the changes of BMD using DXA analysis in three types of fixation up to 2 years post‐operatively	*n* = 90 ‐Mean age = 70 years Palacos = 24 ‐Mean age = 73 years Cemex‐F = 30 ‐Mean age = 71 years Uncemented = 34 ‐Mean age = 65 years	Hip	DXA	T0 = Post surg T1 = 12 m T2 = 24 m	All‐polyethylene cups (Smith & Nephew, Memphis, TN)	Cemented and Cementless	ROI 5 Cup of Hip
Digas, G et al. [24]	International Orthopeadics (2009)	Prospective study	To evaluate the longitudinal changes of BMD during the follow‐up period and to what extent gender, age at operation, weight, side operated, stem size, postoperative BMD and stem subsidence as measured with radiostereometric analysis (RSA) influenced the observed bone remodelling	*n* = 88 ‐Mean age = 60 years Age range 37–78 years Male = 30 Female = 58	Hip	DXA	T0 = Post surg T1 = 12 m T2 = 24 m T3 = 60 m	Spectron Primary, Smith and Nephew, Memphis TN, USA	Cemented	Gruen Zone ROI 7 Femur
Ebert, J et al. [26]	Orthopaedic Surgery, Q2 (2022)	Prospective clinical study	To evaluate the clinical outcome and periprosthetic bone change up until 2 years in a prospective series of patients undergoing primary THA for osteoarthritis with the Absolut cemented stem, together with an investigation of stem migration in a subset of the cohort	*n* = 47 ‐Mean age = 74.2 years ‐Age range = 36–89 years	Hip	DXA	T0 = Post surg T1 = 12 m T2 = 24 m	Absolut femoral stem (Global Orthopaedic Technology Pty Ltd., Sydney, Australia)	Cemented	Gruen Zone ROI 7 Femur
Freitag, T et al. [29]	Arch Orthop Trauma Surg, Q1 (2016)	Prospective randomised study	To evaluate implant‐specific BMD changes during 1‐year follow‐up after THA following short and straight stem implantation	*n* = 138 Fitmore = 57 ‐Mean age = 58.8 years ‐SD = 10.2 years Male = 36 Female = 21 CLS = 81 ‐Mean age 59.1 years ‐SD = 9.3 Male = 52 Female = 31	Hip	DXA	T0 = Post surg (7 d) T1 = 3 m T2 = 12 m	Trochanter‐sparing short stem (Fitmore; Zimmer, Winterthur, Switzerland) + Cementless straight stem (CLS; Zimmer, Winterthur, Switzerland)	Cementless	Gruen Zone ROI 7 Femur
Galli, M et al. [30]	Skelatal Radiology, Q2 (2008)	Prospective cohort study	To evaluate BMD changes around the proximal femur after implantation of two different anatomical stems	*n* = 36 Bihapro = 23 ‐Mean age = 60.9 years Citation = 13 ‐Mean age 59.7 years	Hip	DXA	T0 = Post surg (7 d) T1 = 12 m	Bihapro + Citation stem implant (Howmedica, Rutherford, NJ, USA)		Gruen Zone ROI 7 Femur
Gauthier, L et al. [31]	Hip International, Q2 (2013)	Prospective randomised study	To quantify BMD on the acetabular side with a large‐head MoM bearing and compare it with that of a standard MoP bearing in primary THR	*n* = 50 MoM = 25 ‐Mean age = 60.2 years ‐SD = 7.2 Male = 14 Female = 11 MoP = 25 ‐Mean age = 63.0 years ‐SD = 5.5 Male = 9 Female = 16	Hip	DXA	T0 = Post surg (14 d) T1 = 12 m T2 = 24 m	CONSERVE A‐Class Total Hip System with Big Femoral Head (BFH) technology (Wright Medical Technology, Memphis, Tennessee) + Acetabular system with a highly cross‐linked polyethylene liner (Wrigh Medical Technology)		ROI 4 Peri‐acetabular area
Gazdzik, T et al. [32]	Journal of Clinical Densitometry, Q1 (2008)	Prospective cohort study	To analysis BMD changes at the knee joint arthroplasty site in the course of the first year after surgery	*n* = 106 ‐Mean age = 69.8 years ‐SD = 9.4	Knee	DXA	T0 = Post surg (2w) T1 = 1 m T2 = 3 m T3 = 6 m T4 = 12 m	AGC II Biomet Merck prothesis + PFC Sigma Johnson & Johnson + Scorpio type Stryker prothesis		ROI 4 Tibia + Femur
Gerhardt, D et al. [33]	Hip International, Q2 (2019)	Randomised controlled trial	To compare periacetabular BMD changes between 2 types of MoM hip arthroplasties	*n* = 71 RHA = 38 ‐Mean age = 54.4 years ‐SD = 9.5 THA = 33 ‐Mean age 56.5 years ‐SD = 7.3	Hip	DXA	T0 = Post surg (2w) T1 = 3 m T2 = 6 m T3 = 12 m T4 = 24 m T5 = 36 m T6 = 60 m			ROI 5 Cup of Hip
Grochola, L et al. [34]	Arch Orthop Trauma Surg, Q2 (2008)	Prospective study	To investigate the effect of the stem design on periprosthetic bone remodelling after insertion of an anatomic stem with proximal fixation and the direct comparison to a straight stem prosthesis	*n* = 66 ‐ Mean age = 49.1 years ‐ Age range = 25‐69 years Female = 37 Male = 29 Hip = 68	Hip	DXA	T0 = Post surg (7 d) T1 = 12 m T2 = 24 m	CTX‐S implants + PPF prostheses	Cementless	Gruen Zone ROI 7 Femur
Hayaishi et al. [36]	The Journal of Arthroplasty, Q1 (2007)	Prospective cohort study	To examine whether the Freeman cementless THA, with femoral neck preservation and a large metal head, can prevent stress shielding in a manner similar to resurfacing THA	*n *= 26 Group A = 10 Female = 10 ‐Mean age = 53.0 years ‐SD = 8.0 Group B = 16 Female = 16 ‐Mean age = 61.0 years ‐SD = 11.0	Hip	DXA	T0 = Post surg (3w) T1 = 6 m T2 = 12 m	BHR system (MMT, Birmingham, UK) + BHR Socket and Freeman stem (Finsbury, Surrey, UK)	Cementless	Gruen Zone ROI 7 Femur
Herrera et al. [37]	Journal of Biomechanics, Q1 (2007)	Prospective cohort study Control group	− To analyse the long‐term changes of BMD in the femur after the implantation of ABG‐I − To make two 3D FE models from the scanned geometry corresponding to the healthy femur − To check if the results of the FE simulation make it possible to explain the biomechanical changes	*n* = 61 ‐Mean age = 59.0 years	HIp	DXA	T0 = Post surg T1 = 6 m T2 = 12 m T3 = 36 m T4 = 60 m T5 = 72 m T6 = 120 m	ABG‐I stem (Stryker)	Cementless	Gruen Zone ROI 7 Femur
Herrera et al. [39]	Journal of Arthroplasty, Q1 (2014)	Prospective cohort study Control group	To identify the relationship between changes in bone mass and mechanical stimulus variation, in two cemented stems models, in a mid‐term follow‐up period (five years)	*n* = 64 ‐Mean age = 78.3 years ABG‐II = 32 ‐Mean age = 76.3 years ‐Male = 5 ‐Female = 27 VerSys = 32 ‐Mean age = 72.9 years ‐Male = 5 ‐Female = 27	Hip	DXA	T0 = Post surg (15 d) T1 = 3 m T2 = 12 m T3 = 24 m T4 = 36 m T5 = 48 m T6 = 60 m	ABG‐II (Stryker) + VerSys (Zimmer)	Cemented	Gruen Zone ROI 7 Femur
Huang et al. [41]	Journal of Arthroplasty, Q1 (2013)	Prospective cohort study	To investigate the changes in BMD of acetabulum and proximal femur after total hip resurfacing arthroplasty	*n* = 48 Hip = 51 Group A = 25 ‐Mean age = 46.5 years Male = 11 Female = 14 Hip = 26 Group B = 23 ‐Mean age = 49.0 years Male = 15 Female = 8 Hip = 25	Hip	DXA	T0 = Post surg (2w) T1 = 6 m T2 = 12 m T3 = 24 m T4 = 36 m	Wright Medical Technologies, Arlington, TN + Depuy ASR XL Head system		Gruen Zone ROI 7 Femur
Jahnke, A et al. [43]	International orthopaedics, Q1 (2014)	Case series	To examine the concept of proximal load initiation of a total short‐stemmed hip arthroplasty on the basis of bone variations	*n* = 40 ‐Mean age = 55.4 years Male = 20 Female = 20	Hip	DXA	T0 = Post surg (1w) T1 = 6 m T2 = 12 m	Metha® short‐stem prosthesis	Cementless	Gruen Zone ROI 7 Femur
Kim, Y et al. [48]	The Bone & Joint Journal (2007)	Randomised study	To compare the BMD around cementless acetabular and femoral components which were identical in geometry and had the same alumina modular femoral head, but differed in regard to the material of the acetabular liners	*n* = 50 ‐Mean age = 51.0 years ‐Age range = 35–66 years Male = 38 Female = 12	Hip	DXA	T0: Post surg (1w) T1: 12 m T2: 24 m T3: 36 m T4: 48 m T5: 60 m	Femoral component (IPS, DePuy, Leeds, United Kingdom)	Cementless	Gruen Zone ROI 7 Femur ROI 3 Acetabulum
Kim, Y et al. [46]	The journal of arthroplasty (2011)	Randomised study	To compare BMD Changes Around Short, Metaphyseal‐Fitting, and Conventional Cementless Anatomical Femoral Components	G proxima *n* = 50 ‐Mean age = 54.3 years Male = 22 Female = 28 G Pofile *n* = 50 Mean age = 51.8 years Male = 24 Female = 26	Hip	DXA	T0: Post surg (1w) T1: 3 y	DePuy	Cementless	Gruen Zone ROI 2 Femur
Kim, Y et al. [47]	Clinical Orthopaedics and Related Research, Q1 (2014)	Retrospective cohort study	To evaluat long‐term clinical results using validated scoring instruments; osseointegration and bone remodelling; complications; and rates of revision and osteolysis in patients younger than 65 years who underwent THA with a short, metaphyseal‐fitting anatomic cementless stem	*n* = 500 ‐Mean age = 52.7 years Male = 314 Female = 186	Hip	DXA	T0: Post surg (1w) T1: 15.8 y	Short, metaphyseal‐fitting anatomic cementless stem	Cementless	Gruen Zone ROI 7 Femur ROI 3 Acetabulum
Koppens, D et al. [49]	The Journal of Arthroplasty (2020)	RCT	To examine the influence of systemic and periprosthetic BMD on migration of the tibial component of cemented medial UKA with 2 years follow‐up	*n* = 65	Knee	DXA	T0: Post surg (1w) T1: 4 M T2: 12 M T3: 24 M	Mobile‐bearing (MB) UKA (Oxford Partial Knee; Zimmer Biomet, Bridgend, UK) + Fixed‐bearing (FB) UKA (Sigma High Performance Partial Knee System; DePuy International Ltd, Leeds, UK)		ROI 4 Tibia
Leichtle, U et al. [53]	The Bone & Joint Journal (2006)	Prospective cohort study	To investigate the clinical results related to the bony integration of a femoral component in the medium term, as well as the peri‐prosthetic bone remodelling processes, over approximately a five‐year period after surgery	*n* = 43 ‐Mean age = 54 years Male = 24 Female = 19	Hip	DXA	T0: Post surg (8 d) T1: 3 M T2: 6 M T3: 3.6 y T4: 4.6 y	Evolution K (Fehling Medical AGI, Karlstein, Germany) + Harris‐Galante acetabular component.	Cementless	Gruen zone ROI 7 Femur
Lerch, M et al. [55]	Journal of orthopaedic research, Q1 (2012)	Prospective investigation	To answer the following research questions: (i) what is the effect of THA with the Metha® short stem on femoral bone remodelling?; (ii) can numerical computations be confirmed by DXA measurement of bone remodelling?; and (iii) what are the differences and can we explain them?	*n* = 25 ‐Mean age = 58.9 years Male = 16 Female = 9	HIp	DXA	T0: Post surg (1w) T1: 6 M T2: 1 y T3: 2 y	Bicontact® total hip arthroplasty system (AESCULAP AG, Tuttlingen, Germany) + Plasmacup SC press‐fit acetabular component or the SC‐Screwcup (both BBraun, Aesculap, Tuttlingen, Germany)	Cementless	Gruen Zone ROI 7 Femur
Liu, Y et al. [58]	Orthopaedic Surgery, Q2 (2022)	Retrospective study	To compare the periprosthetic BMD changes around Tri‐Lock 'Bone Preserving Stem' with the other two common and longer stems (Corail and Summit) after THA	*n* = 138 Tri‐Lock stem = 49 Corail stem = 44 Summit stem = 45	Hip	DXA	T0 = Post surg (1w) T1 = 5 y	Tri‐Lock BPS stem (Depuy, Eagan, MN, USA) + Corail stem (DePuy Synthes, Raynham, MA, USA) + Summit stem (Depuy Orthopedics, Inc., Warsaw, In, USA)	Cementless	Gruen Zone ROI 7 Femur
López‐Subías, J et al. [60]	Journal of Clinical Densitometry, Q2 (2019)	Prospective study	To establish the pattern of bone remodelling caused by a cementless, and anatomic implant	*n* = 37 ‐Mean age = 57.3 years ‐Age range: 36–75 years Male = 31 Female = 6	Hip	DXA	T0 = Post surg T1 = 3 m T2 = 6 m T3 = 1 y	ANATO® stem (Stryker®, USA)	Cementless	Gruen Zone ROI 7 Femur
MacDonald, S et al. [61]	Clinical Orthopaedics and Related Research, Q1 (2010)	Randomised controlled trial	To examine differences in clinical scores, incidence of thigh pain, and development of stress shielding	*n* = 388 Synergy™ = 198 ‐Mean age = 61 years Prodigy™ = 190 ‐Mean age = 60 years	Hip	DXA	T0 = Post surg (2w) T1 = 6 m T2 = 1 y T3 = 2 y	Tapered, titanium, proximally porous‐coated (titanium bead) stem (SynergyTM; Smith and Nephew Inc, Memphis, TN) + Cylindrical, cobalt‐chrome, fully porous‐coated (cobaltchrome‐molybdenum alloy bead) stem (ProdigyTM; DePuy Inc, Warsaw, IN)	Cementless	Gruen Zone ROI 7 Femur
Merle, C et al. [62]	Sage journals (2012)	Comparative longitudinal study	To determine the extent and the pattern of femoral periprosthetic bone remodelling following uncementened THA around straight, double‐tapered, grit‐blasted titanium stems comparing a muscle sparing anterolateral surgical approach to a muscle detaching transgluteal surgical approach	Group A (anterolateral) = 16 ‐Mean age = 63 years Male = 6 Female = 10 Group B (transgluteal) = 26 ‐Mean age = 58 years Male = 14 Female = 12	Hip	DXA	T0 = Post surg T1 = 3 m T2 = 6 m T3 = 1 m	CLS stem (Zimmer, Warsaw, USA)	Cementless	Gruen zone ROI 7 Femur
Meyer, J et al. [63]	Journal of Clinical Densitometry: assessment & management of muscoloskeletal health (2018)	Prospective randomised DXA‐analysis	To evaluate the implant‐specific femoral BMD changes 5 yr after THA, comparing a cementless bone preserving stem (Fitmore, Zimmer Biomet, Warsaw, IN) and a cementless straight stem (CLS Spotorno, Zimmer Biomet, Warsaw, IN), using DXA	Fitmore short stem = 57 ‐Mean age = 56.8 years Male = 36 Female = 21 CLS straight stem = 83 ‐Mean age = 59.1 years Male = 52 Female = 31	Hip	DXA	T0 = Post surg (7 d) T1 = 12 m T2 = 60 m	Fitmore, Zimmer Biomet, Warsaw, IN + CLS Spotorno, Zimmer Biomet, Warsaw, IN	Cementless	Gruen zone ROI 7 Femur
Meyer, J et al. [64]	Orthopaedics & Traumatology: surgery & research, Q1 (2020)	Prospective randomised study without control group	To evaluate if there is an influence of gender on implant‐specific stress shielding after implantation of a curved bone preserving hip stem (Fitmore, Zimmer Biomet, Warsaw, IN, USA) 5 years postoperatively	*n* = 57 Male = 37 ‐ Mean age = 59.3 ± 8 years Female = 20 ‐ Mean age = 55.4 ± 11.2 years	Hip	DXA	T0 = Post surg (7 d) T1 = 12 m T2 = 60 m	Fitmore, Zimmer Biomet, Warsaw, IN + CLS Spotorno, Zimmer Biomet, Warsaw, IN	Cementless	Gruen Zone ROI 7 Femur
Minoda, Y et al. [65]	Knee Surgery, Sports Traumatology, Arthroscopy, Q1 (2022)	Prospective comparative stud	To determine whether the advantage of mobile‐bearing TKA over conventional fixed‐bearing TKA changes even at a mean of 11 years postoperatively	Mobile‐bearing prosthesis = 28 Fixed‐bearing prosthesis = 28	Knee	DXA	T0 = Post surg (2w) T1 = 3 m T2 = 6 m T3 = 12 m T4 = 18 m T5 = 24 m T6 = 5 y then annually thereafter	Fixed‐bearing posterior stabilised (PS) prosthesis (NexGen LPS‐Flex; Zimmer Biomet, Warsaw, IN, USA) + Mobile‐bearing PS prosthesis (P.F.C. Sigma RP; DePuy Synthes, Raynham, MA, USA)	Cemented	ROI 3 Femur
Minoda, Y et al. [66]	The Knee, Q2 (2022)	Prospective cohort study	To compare the peg position and BMD around the peg in a cementless porous tantalum tibial component after TKA using the same study population of our previous report	*n* = 27 ‐Mean age = 74 ± 7 years Male = 6 Female = 21	Knee	DXA	T0 = Post surg (2w) T1 = 1 y T2 = 2 y	Porous tantalum tibial component (Trabecular metal monoblock tibial component; Zimmer) + Fixed bearing posterior stabilised prosthesis (NexGen LPS‐Flex; Zimmer)	Cementless	ROI 3 Tibia
Minoda, Y et al. [67]	The journal of arthroplasty (2013)	Matched cohort study	To compare the BMD in the proximal part of the tibia between TKA using a porous tantalum tibial component than that using a conventional cemented cobalt‐chromium tibial component for 5 years	Trabecular metal group = 21 ‐Mean age = 72.6 ± 6.7 years Male = 4 Female = 18 Cemented group = 21 ‐Mean age = 71.1 ± 6.3 years Male = 5 Female = 17	Knee	DXA	T0 = Post surg (2w) T1 = 3 y T2 = 4 y T3 = 5 y	Porous tantalum tibial component and cemented cobalt‐chromium femoral component (NexGen LPS‐Flex; Zimmer) + Cemented cobalt‐chromium‐alloy tibial component (P.F.C. Sigma RP; DePuy, Warsaw, IN)	Cemented	ROI 3 Tibia
Minoda, Y et al. [68]	The journal of arthroplasty (2020)	Clinical Trial	To update a matched cohort study at a minimum of 6 years' follow‐up period	Trabecular metal group = 20 ‐Mean age = 72.4 ± 6.5 years Male = 2 Female = 18 Cemented group = 18 ‐Mean age = 70.7 ± 6.7 years Male = 5 Female = 13	Knee	DXA	T0 = Post surg (2w) T1 = 1 y T2 = 5 y T3 = 11 y	Porous tantalum tibial component and a cemented cobaltchromium femoral component (NexGen LPS‐Flex; Zimmer) + Cemented cobalt‐chromiumalloy tibial component (P.F.C. Sigma RP; DePuy, Warsaw, IN),	Cemented	ROI 3 Tibia
Morita, D et al. [74]	Journal of Orthopaedic Science, Q2 (2016)	Prospective study	To prospectively quantify longitudinal changes in BMD for more than 3 years after the insertion of a cemented Exeter universal stem and determine the extent to which gender, age at surgery, weight, height, body mass index (BMI), surgical side, stem subsidence, and Japanese Orthopaedic Association (JOA) score affected these changes	*n* = 150 Hip = 165 Male = 20 Female = 130	Hip	DXA	T0 = Post surg (2w) T1 = 3 y	Stryker Orthopaedics, Mahwah, New Jersey, USA	Cemented	Gruen Zone ROI 7 Femur
Motomura, G et al. [76]	Scientific reports, Q1 (2022)	Multicenter randomised controlled study	To compare stems with a porous tantalum surface versus a titanium fibre mesh surface stem in terms of periprosthetic bone remodelling	*n* = 118 Male = 11 Female = 107 Trabecular metal = 59 ‐Mean age = 62.1 ± 8.5 years Male = 4 Female = 55 VerSys = 59 ‐Mean age = 60.9 ± 8.0 years Male = 7 Female = 52	Hip	DXA	T0 = Post surg (1w) T1 = 6 m T2 = 12 m T3 = 24 m	Trabecular Metal Primary Hip Prosthesis; Zimmer‐Biomet, Warsaw, IN + VerSys HA‐TCP Fibre Metal Taper Stem; Zimmer‐Biomet	Cementless	Gruen Zone ROI 7 Femur
Nysted, M et al. [77]	Acta Orthopaedica, Q1 (2011)	Prospective comparative study	To compare the medium‐term changes in BMD in the proximal femur after insertion of an uncemented, customised femoral stem and an uncemented, standard anatomical femoral stem	*n* = 87 Male = 31 Female = 56 ABG‐I femoral stem = 41 Unique femoral stem = 46	Hip	DXA	T0 = Post surg T1 = 3 m T2 = 6 m T3 = 12 m T4 = 24 m T5 = 36 m T6 = 60 m	SCP, Trondheim, Norway + Stryker‐Howmedica, Allendale, NJ	Cementless	Gruen Zone ROI 7 Femur
Nyström, A et al. [78]	Acta Orthopaedica, Q1 (2022)	Prospective cohort study	To examine the long‐term changes in periprosthetic BMD and stability of the CFP stem	*n* = 21 ‐Mean age = 64 years ‐Age range = 55–73 years Male = 11 Female = 10	Hip	DXA	T0 = Post surg (2 d) T1 = 1 y T2 = 2 y T3 = 8 y	Uncemented CFP stem + Uncemented trabeculae‐oriented pattern (TOP) cup (Waldemar Link GmbH & vCo. KG, Hamburg, Germany)	Cementless	Gruen Zone ROI 7 Femur
Panisello, J et al. [80]	International Orthopaedics (2009)	Prospective cohort study	To quantify the effect that a thinner, shorter and polished diaphyseal part of the stem had on promoting better metaphyseal load transfer by analysing the BMD changes in the proximal femur	ABG‐I group = 56 ‐Mean age = 60.1 years ‐Age range = 39–85 years Male = 27 Female = 29 ABG‐II group = 54 ‐Mean age = 59,2 years ‐Age range = 38–83 years Male = 26 Female = 28	Hip	DXA	ABG‐I stem T0 = Post surg T1 = 6 m T2 = 1 y T3 = 10 y ABG‐II stem T0 = Post surg (15 d) T1 = 6 m T2 = 1 y T3 = 5 y	ABG‐I stem + ABG‐II stem	Cementless	Gruen Zone ROI 7 Femur
Panisello, J et al. [81]	The Journal of Arthroplasty (2009)	Prospective and controlled study	‐To determine the pattern of remodelling produced by this stem ‐To quantify the changes of BMD in the 7 zones of Gruen throughout the follow‐up ‐To prove or reject the presence of positive long term remodelling ‐To quantify the effect of aging on periprosthetic BMD	*n* = 61	Hip	DXA	T0 = Post surg T1 = 6 m T2 = 1 y T3 = 10 y	ABG‐I stem (Stryker, Howmedica)	Cementless	Gruen Zone ROI 7 Femur
Pitto, R et al. [82]	International Orthopedics (2008)	Prospective study	To assess femoral bone adaptive remodelling around an uncemented femoral component with a taper design and hydroxyapatite (HA) coating	*n* = 29 ‐ Mean age = 58 years ‐Age range = 30–80 years Male = 16 Female = 13 Hip = 32	Hip	qCT	T0 = Post surg T1 = 1 y T2 = 2 y	THA with a taper‐design femoral component coated with HA (Summit; DePuy International, Leeds, UK) + Press‐fit titanium cup (Duraloc; DePuy) with alumina‐alumina pairing (Biolox, CeramTec, Plochingen, Germany)	Cementless	2 mm slice ROI 5 Femur
Pitto, R et al. [83]	International Orthopedics (2010)	Prospective study one‐cohort	To assess femoral bone adaptive remodelling around an uncemented femoral component with a taper design and hydroxyapatite (HA) coating five years after the index operation	*n* = 29 ‐Mean age = 58 years Male = 16 Female = 13 Hip = 31 hips	Hip	qCT	T0 = Post surg T1 = 1 y T2 = 2 y T3 = 5 y	THA with a taper‐design femoral component coated with HA (Summit; DePuy International, Leeds, UK) + Press‐fit titanium cup (Duraloc; DePuy) with ceramic−ceramic pairing (Biolox Delta, CeramTec, Plochingen, Germany)	Cementless	2 mm slice ROI 5 Femur
Rathsach Andersen, M et al. [84]	Acta Orthopedica, Q1 (2019)	Randomised controlled trial	To quantify bone remodelling of the proximal tibia after implantation of the Trabecular Metal Technology (TMT) Zimmer Nexgen	*n* = 70 Age < 70	Knee	DXA	T0 = Post surg (1w) T1 = 3 m T2 = 6 m T3 = 12 m T4 = 24 m	Zimmer Nexgen Flex	Cementless	ROI 3 Tibia
Saari, T et al. [88]	Journal of Orthopaedic research (2007)	Randomised trial	To compare BMD changes in Resection vs retention of PCL in TKA	PCL retained and flat insert ‐Mean age = 69 years ‐Age range = 51–77 years Male = 1 Female = 12 PCL retained and concave insert ‐Mean age = 66 years Age range = 59–79 years Male = 3 Female = 8 PCL resected and concave insert ‐Mean age = 69 years Age range = 50–82 years Male = 4 Female = 11 PCL resected and posterior stabilised insert‐Mean age = 78 years ‐Age range = 55–81 years Male = 3 Female = 4	Knee	DXA	T0 = Post surg (7 d) T1 = 1 y T2 = 2 y T3 = 5 y	AMK TKR (DePuy; Johnson & Johnson, Leeds, UK)	Cemented	ROI 3 Tibia
Soininvaara, T et al. [90]	Clinical Physiology and functional imaging, Q2 (2008)	Comparative study	To investigate early regional periprosthetic BMD changes in comparison with metabolic activity detected by single photon emission computed tomog‐raphy (SPECT)	*n* = 16 ‐Mean age = 66 years Male = 5 Female = 11	Knee	DXA	T0 = Post surg T1 = 6 m T2 = 1 y T3 = 2 y	Duracon modular (Howmedica Inc. Rutherford, NJ⁄International Division of Pfizer) + Nexgen (Zimmer, Warsaw, IN, USA) + AMK (DePuy, Division of Boehringer Mannheim Corporation⁄DePuy, Warsaw, IN, USA).	Cemented	ROI 3 Tibia ROI 3 Femur
Soininvaara, T et al. [91]	The knee, Q1 (2013)	Prospective case control study	To determine whether UKA preserves periprosthetic BMD, particularly in the femoral regions	*n* = 21 ‐Mean age = 65.2 years Male = 8 Female = 13	Knee	DXA	T0 = Post surg (7 d) T1 = 3 m T2 = 6 m T3 = 1 y T4 = 2 y T5 = 4 y T6 = 7 y	Duracon unicondylar (Howmedica International Inc., Division of Pfizer Hospital Product Group, Shannon Industrial Estate, Ireland) + Miller‐Galante (Zimmer, Warsaw, IN, USA)		ROI 3 Tibia ROI 5 Femur ROI 1 Patella
Steens, W et al. [92]	BMC Musculoskeletal Disorders, Q1 (2015)	Prospective study one‐cohort	To prospectively investigate the in vivo changes of BMD as a parameter of bone remodelling around a short, femoral neck prosthesis over the first 5 years following implantation	*n* = 20 Male = 12 Female = 8	Hip	DXA	T0 = Post surg (10 d) T1 = 3 m T2 = 12 m T3 = 60 m	“Stemless” ESKA CUT 2000 femoral neck prosthesis (ESKA Orthodynamics, Luebeck, Germany)	Cementless	Gruen Zones ROI 7 Femur
Stilling, M et al. [94]	Orthopaedic Surgery, Q4 (2012)	Randomised controlled trial	To present preliminary clinical and radiological results at 6 months follow‐up after Copeland and Global Cap RHHI	*n* = 21 ‐Mean age = 64 years ‐Age range = 39–82 years Male = 11 Female = 10 Copeland group = 10 ‐Mean age = 66 years ‐Age range = 40–82 years Male = 6 Female = 3 Global C.A.P. group = 11 ‐Mean age = 61 years ‐Age range = 53–83 years Male = 4 Female = 6	Shoulder	DXA	T0 = Post surg T1 = 6 m	Copeland (Biomet Inc.) + Global C.A.P. (DePuy Int)	Cementless	ROI 1 Humerus
Synder, M et al. [96]	Orthopedics, Q1 (2015)	Prospective study	To evaluate early bone remodelling around the Metha stem during 12 months of follow‐up	*n* = 36 ‐Mean age = 50.4 years Male = 18 Female = 18	Hip	DXA	T0 = Post surg (10 d) T1 = 3 m T2 = 6 m T3 = 12 m	Metha stem		Gruen Zone ROI 7 Femur
Tapaninen, T et al. [97]	Scandinavian Journal of Surgery, Q2 (2012)	Clinical trial	To study the BMD changes 3 and 12 months after RHA	*n* = 26 ‐Mean age = 55.2 years Male = 22 Female = 4	Hip	DXA	T0 = Post surg T1 = 3 m T2 = 1 y	Birmingham hip resurfacing system (Smith & Nephew UK, London, WC2N 6LA, UK.) + Conserve (plus) (Wright Medical Technology, Inc. Arlington, TN 38002, USA) + Cormet (Stryker, Kalamazoo, MI 49002, USA) + Biomet Recap (Biomet, Inc. Warsaw, Indiana, 46581‐0587, USA)		Gruen Zone ROI 4 Femural neck
ten Broeke, R et al. [14]	Hip international, Q1 (2012)	Randomised clinical trial	To compare bone remodelling around two uncemented stems	Symax stem = 25 Omnifit‐HA = 24	Hip	DXA	T0 = Post surg (1w) T1 = 6w T2 = 3 m T3 = 6 m T4 = 1 y T5 = 2 y	SymaxTM (n = 25) + Omnifit® (n = 24) stems	Cementless	Gruen Zone ROI 7 Femur
Venesmaa, P et al. [100]	Acta ortopedica scandinava (2003)	Prospective study	To eter‐mined the periprosthetic BMD change in femoral bone after cemented THA over a 5‐year period	*n* = 17 ‐Mean age = 68 years Male = 7 Female = 10	Hip	DXA	T0 = Post surg (2w) T1 = 3 m T2 = 6 m T3 = 1 y T4 = 2 y T5 = 3 y T6 = 5 y	Cobalt‐chrome Lubinus SPII stems with a collar (Waldemar Link MBH&CD, Germany)	Cemented	Gruen Zone ROI 7 Femur
Vidovic, D et al. [101]	Injury, Q1 (2013)	Randomised clinical trial	To evaluate the magnitude of BMD as well as the clinical results after cemented and cementless haemiarthroplasty (HA) for femoral neck fracture	*n* = 60 Cemented group A = 30 ‐Mean age = 82.90 ± 4.63 years Uncemented group B = 30 ‐Mean age = 82.04 ± 4.32 years	Hip	DXA	T0 = Post surg (1 m) T1 = 3 m T2 = 6 m T3 = 1 y	Cemented + Cementless haemiarthroplasty (HA)	Cemented and Cementless	Gruen Zone ROI 7 Femur
Winther, N et al. [105]	International orthopaedics, Q1 (2015)	Randomised controlled trial	To evaluate the adaptive bone remodelling of the proximal tibia after uncemented TKA using a tibial tray with Regenerex coating compared to a well‐proven standard porous coated (PPS) tibial tray	*n* = 61 Regenerex = 31 ‐Mean age = 63 years Male = 16 Female = 15 Porous plasma = 30 ‐Mean age = 62 years Male = 11 Female = 19	Knee	DXA	T0 = Post surg (1w) T1 = 3 m T2 = 6 m T3 = 12 m T4 = 24 m	Vanguard PPS (Biomet, Warsaw, Indiana, USA) + Vanguard Regenerex Primary Tibial Tray (Biomet, Warsaw, Indiana, USA)	Cementless	ROI 3 Tibia
Zerahn, B et al. [109]	Hip International, Q1 (2011)	Prospective randomised study	To assess whether different bearing materials have an impact on femoral bone remodelling within the first four years after a hybrid THA	*n *= 398 Group A: Zirconia ceramic head, polyethylene cup = 97 Group B: Cobalt‐Chrome‐Molybdenum head and cup = 88 Group C: Zirconia ceramic head, polyethylene moulded on the Titanium shell of the Asian cup = 122 Group D: Alumina head and cup = 91	Hip	DXA	T0 = Post surg (1w) T1 = 4 y	Universal RingLoc Ti6Al4V‐alloy (Biomet, Warsaw, Indiana, USA)	Cemented	Gruen Zones ROI 7 Femur

Abbreviations: AMK, anatomic modular knee; AML, anatomic medullary locking; BFH, big femoral head; BMD, bone mineral density; BMI, body mass index; d, day; DXA, dual‐energy X‐ray absorptiometry; FB, fixed‐bearing; HA, hydroxyapatite; m, month; MB, mobile‐bearing; PCL, posterior cruciate ligament; PPS, standard porous coated; PS, posterior stabilised; RHA, resurfacing hip arthroplasty; ROI, region of interest; Surg., surgery; THA, total hip arthroplasty; TKA, total knee arthroplasty; TMT, trabecular metal technology; TOP, trabeculae‐oriented pattern; UKA, unicompartmental knee arthroplasty; w, week.

Among the articles included, 55 articles analysed the hip joint, of which 45 used the standard 7 Gruen zones to determine the ROIs around the femoral component, while five, four, and two ROIs were evaluated by three, two and one studies respectively.

The acetabulum was investigated in seven studies, the majority (4) considered three ROIs, two papers inspected five ROIs and four ROIs were studied in one publication. Three articles analysed both the acetabulum and the femur with four and seven ROI, respectively.

Since there is no standardised description of periprosthetic ROIs in total knee arthroplasty, the 12 articles [4, 32, 49, 65, 66, 67, 68, 84, 88, 90, 91, 105] that investigated this joint measured BMD in different regions. The tibia was evaluated in 8 studies, the femur in four studies and two articles investigated both districts. Unicompartmental knee arthroplasty (UKA) was the topic of interest of two included articles, one of which assessed the tibial, femoral and patellar periprosthetic bone, while the other only the tibia.

Furthermore, concerning the fixation technique used for the implant, 16 studies evaluated cemented hips, 42 cementless hip implants, four both cemented and cementless. Cemented knee implants were evaluated in eight studies and six papers assessed cementless knee prosthesis. Two analysed both cementless and cemented knee prosthesis.

Only one article measured BMD variation after total shoulder replacement was found.

### Risk of bias in studies

A total of 68 articles were reviewed, and out of these, 16 were evaluated using ROB2. Eleven of the 16 articles had a moderate risk of bias, and five of the 16 had a high risk. The JBI evaluated the other articles. The JBI Critical Appraisal Checklist for Analytical Cross‐Sectional Studies was used to evaluate a total of 32 articles; the JBI Critical Appraisal Checklist for Cohort Studies was used to evaluate 17 articles; the JBI Critical Appraisal Checklist for Case Series was used to evaluate one article; and the JBI Critical Appraisal Checklist for Quasi‐Experimental Studies was used to evaluate two articles. In supplementary material (Supporting Information: Annex A ‐ Table S2‐5), the methodological quality's results are documented.

### Total hip arthroplasty

A comparison of the weighted mean BMD among the articles over time was performed. Concerning the femoral component, only studies with hip replacement that used the standard Gruen Zone (seven ROIs) method for the femoral periprosthetic BMD evaluation and that reported the results in g/cm^2^ were taken into consideration. Consequently, a total of 3473 hips were included in this analysis. The evaluations performed within the first month of surgery were considered as baseline. All the absolute values of BMD and percentage differences between the baseline and follow‐up are reported in Supporting Information: Annex A ‐ Tables S7‐S8.

To conduct our analysis, only follow‐up periods with over 800 patients, which corresponded to 3, 6, 12, 24 and 60 months, were considered (Table 2). The overall baseline BMD in THA was 1.49 g/cm^2^ considering all seven Gruen zones, and 1.04 g/cm^2^ in the acetabular ROIs (Supporting Information: Annex A ‐ Table S9). The negative peak of BMD decrease has been observed at 6‐month follow‐up (−7.6% from baseline), then BMD increased at 60 months (0.4%).

**Table 2:** Bone mineral density after THA.

ROI	Post surg	3 m		6 m		12 m		24 m		60 m	
	Mean (g/cm^2^)	Mean (g/cm^2^)	Difference (%)	Mean (g/cm^2^)	Difference (%)	Mean (g/cm^2^)	Difference (%)	Mean (g/cm^2^)	Difference (%)	Mean (g/cm^2^)	Difference (%)
1	0.90	0.83	−7.8%	0.72	−19.3%	0.78	−12.7%	0.83	−7.7%	0.81	−9.7%
2	1.55	1.48	−4.5%	1.42	−8.2%	1.51	−2.7%	1.55	0.0%	1.52	−1.5%
3	1.74	1.83	5.1%	1.67	−4.4%	1.82	4.6%	1.79	2.7%	1.85	6.2%
4	1.75	1.87	7.1%	1.71	−2.1%	1.84	5.2%	1.84	5.4%	1.84	5.3%
5	1.79	1.84	3.2%	1.72	−3.5%	1.85	3.4%	1.83	2.7%	1.87	4.7%
6	1.51	1.48	−2.3%	1.41	−7.2%	1.48	−2.1%	1.51	−0.5%	1.51	−0.4%
7	1.21	1.06	−12.2%	1.00	−17.8%	1.05	−13.3%	1.05	−13.1%	1.09	−10.2%
Mean	1.49	1.48	−0.5%	1.38	−7.6%	1.48	−1.1%	1.49	−0.4%	1.50	0.4%
Hip (n°)	3473	898		1383		2255		1277		836	

Abbreviations: ROI, region of interest; THA, total hip arthroplasty.

**Figure 2:**
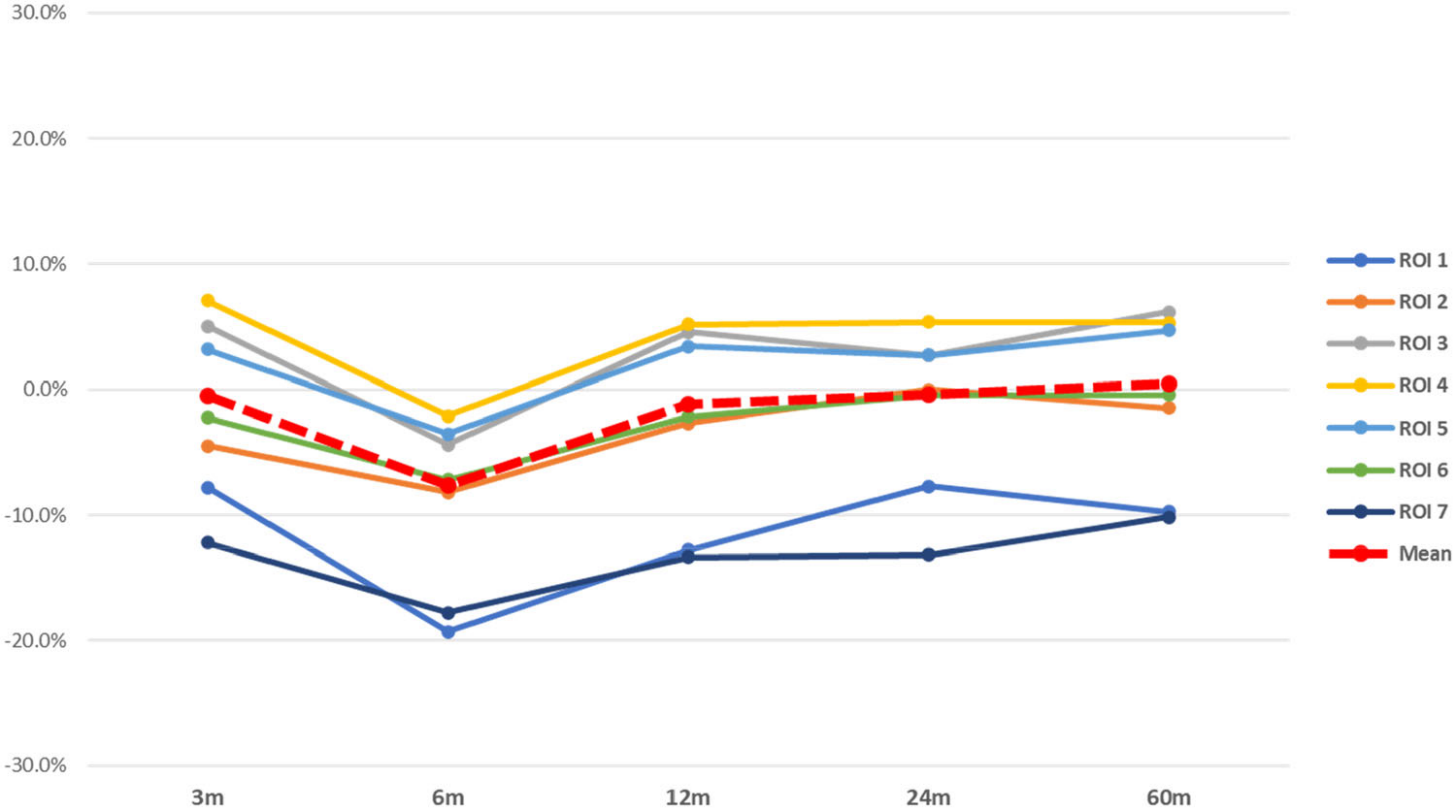
Overall BMD variation (%) after THA. BMD, bone mineral density; ROI, region of interest; THA, total hip arthroplasty.

Considering the variation of each Gruen zone, ROI 1 (equivalent to the greater trochanter) and 7 (equivalent to the calcar region) registered the greatest BMD decrease at every follow up measurement, with a nadir at 6 months and at 60 months. Conversely, ROIs 3, 4, 5 and 6 showed a different trend, with a decrease of BMD at 6 months, whereas, at 60 months, they registered an increase from baseline (Figure 2).

### Fixation technique in THA

**Figure 3:**
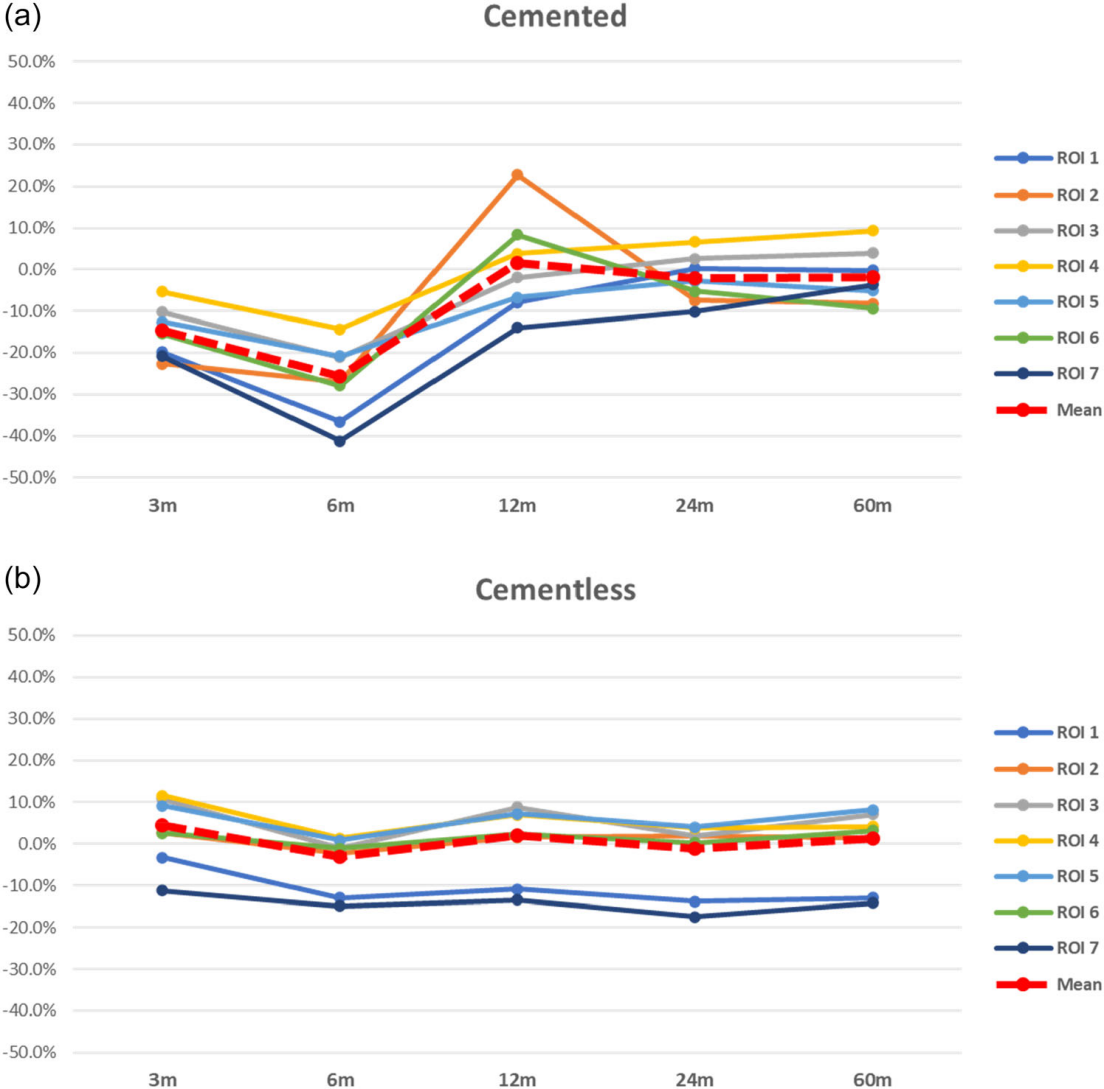
Femoral bone mineral density comparison between cemented and cementless stems in THA across ROIs between the baseline and Follow‐up times. *Note:* (a) Cemented stems BMD. (b) Cementless stems BMD. BMD, bone mineral density; ROI, region of interest; THA, total hip arthroplasty.

A total of 813 cemented hips and 2660 cementless femoral implants were included in this analysis (Table 3). The comparison of the weighted means showed a greater average BMD decrease in cemented compared to cementless implants at 60 months follow‐up. Furthermore, cemented implants showed a considerable BMD decrease compared to cementless stems at 3 and 6‐months follow‐up. However, an increased bone resorption was observed at the level of the proximal femur in both designs, with a more pronounced decrease in cementless stems (Figure 3).

**Table 3:** Comparison of BMD variation (%) between cemented and cementless hip replacement between the baseline and follow‐up times.

ROI	Cemented					Cementless				
	3 m	6 m	12 m	24 m	60 m	3 m	6 m	12 m	24 m	60 m
1	−19.8%	−36.6%	−7.9%	0.2%	−0.3%	−3.3%	−12.9%	−10.8%	−13.7%	−12.9%
2	−22.7%	−27.0%	22.9%	−7.4%	−8.1%	2.8%	−2.2%	1.6%	2.0%	1.3%
3	−10.3%	−21.0%	−2.0%	2.5%	3.9%	10.8%	−1.0%	8.8%	2.0%	7.1%
4	−5.4%	−14.3%	3.8%	6.6%	9.3%	11.6%	1.4%	7.0%	3.8%	4.2%
5	−12.6%	−20.8%	−6.7%	−2.8%	−5.2%	9.1%	0.8%	7.3%	4.1%	8.3%
6	−15.4%	−28.0%	8.4%	−5.3%	−9.4%	2.6%	−1.0%	2.3%	0.3%	3.2%
7	−20.8%	−41.2%	−13.9%	−10.1%	−3.6%	−11.2%	−14.9%	−13.4%	−17.5%	−14.1%
Mean	−14.7%	−25.6%	1.6%	−2.1%	−1.8%	4.5%	−3.1%	2.0%	−1.2%	1.2%
Hip (n°)	241	86	326	358	187	657	1297	1929	919	649

Abbreviations: BMD, bone mineral density; ROI, region of interest.

### Implant design in THA

**Figure 4:**
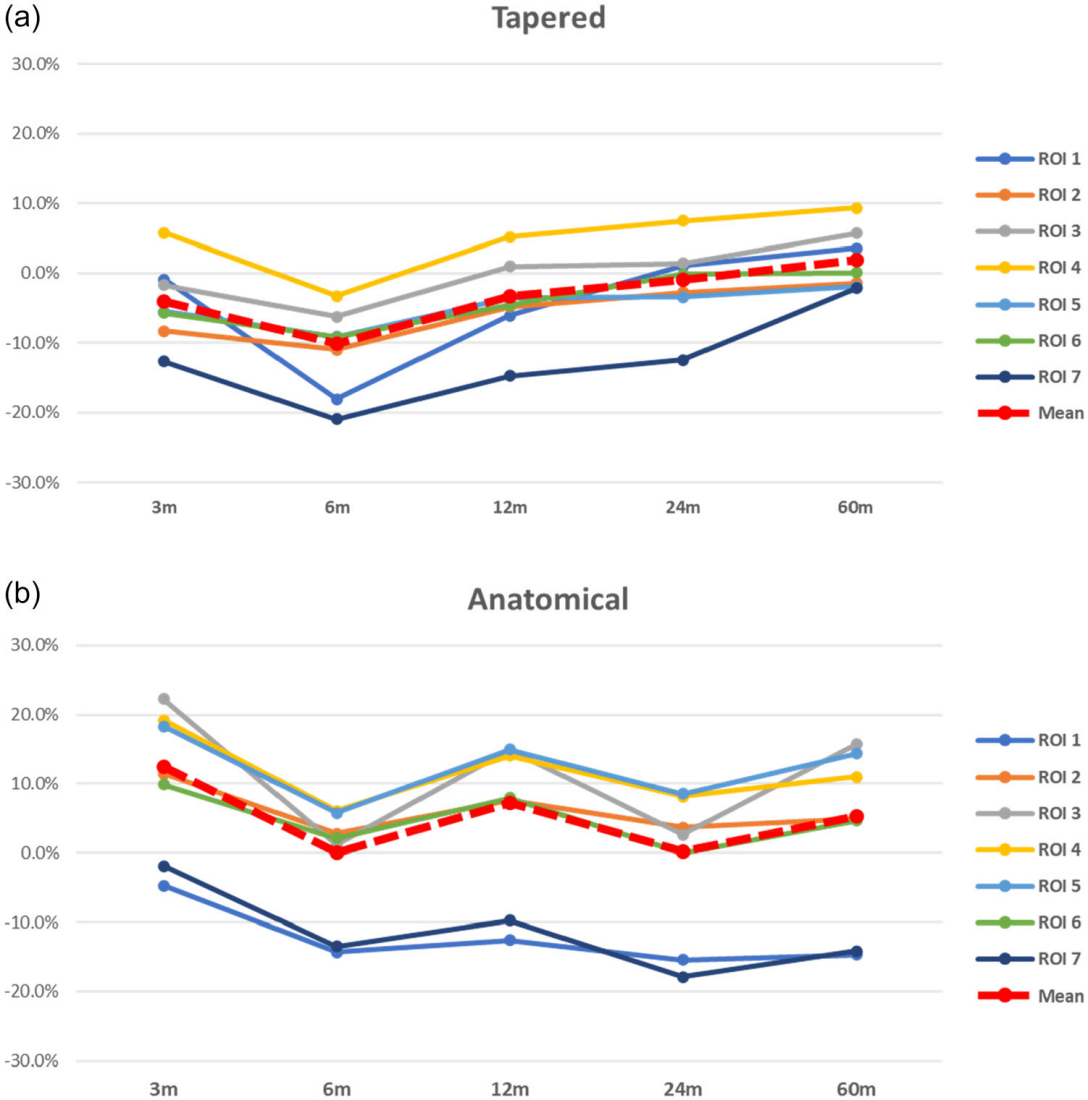
Femoral BMD comparison between stem designs THA across ROIs between the baseline and Follow‐up times. *Note:* (a) Tapered stems. (b) Anatomical stems. BMD, bone mineral density; ROI, region of interest; THA, total hip arthroplasty.

Concerning the THA stem design, a total of 1187 tapered and 1646 anatomic stems were analysed (Table 4). Tapered stems showed a greater average BMD decrease at each follow‐up compared to anatomic stems. The greatest bone resorption was recorded at 6 months of follow‐up at the level of the proximal femur in both designs, however tapered stems showed a greater decrease in BMD than anatomical ones. At 60 months follow‐up the anatomical stems recorded a considerable loss of BMD at the level of the proximal femur (ROI 1 and 7), whereas tapered stems showed a more uniform distribution of bone loss around the stem (Figure 4).

**Table 4:** Comparison of BMD between tapered and anatomic designs in Hip.

ROI	Tapered					Anatomic				
	3 m	6 m	12 m	24 m	60 m	3 m	6 m	12 m	24 m	60 m
1	−0.9%	−18.1%	−6.1%	1.1%	3.6%	−4.7%	−14.3%	−12.5%	−15.4%	−14.7%
2	−8.3%	−11.0%	−4.8%	−2.8%	−1.4%	11.5%	2.8%	7.5%	3.7%	4.9%
3	−1.7%	−6.2%	1.0%	1.4%	5.7%	22.3%	1.2%	14.9%	2.6%	15.7%
4	5.7%	−3.2%	5.2%	7.5%	9.3%	19.2%	6.0%	14.1%	8.1%	11.0%
5	−5.5%	−9.1%	−3.5%	−3.4%	−1.9%	18.3%	5.8%	14.9%	8.5%	14.4%
6	−5.7%	−9.2%	−4.6%	−0.1%	0.1%	9.9%	2.1%	8.0%	0.0%	4.6%
7	−12.7%	−21.0%	−14.7%	−12.5%	−2.1%	−1.8%	−13.5%	−9.7%	−17.9%	−14.1%
Mean	−4.0%	−10.1%	−3.2%	−1.0%	1.9%	12.5%	0.0%	7.3%	0.2%	5.2%
Hip (n°)	326	515	763	708	311	434	602	1053	456	315

Abbreviations: BMD, bone mineral density; ROI, region of interest.

### Acetabular component

A total of seven studies including 609 cups measured periprosthetic acetabular bone density. A standardised description of the ROIs around the acetabular cup was described by DeLee and Charnley, who identified three regions: lateral, central and medial [23]. Nevertheless, four studies among them used these ROIs [28, 41, 47, 48], while Digas et al. [25] and Gerhardt et al. [33] analysed five ROIs, and Gauthier et al. four ROIs [31].

The authors who utilised DeLee and Charnley's zones observed the following pattern: BMD in ROI 1 (lateral) increased from baseline to 6, 12, 24 and 60 months. A similar behaviour was evident in ROI 2 (central) with a smaller increase. Whereas BMD in ROI 3 (medial) showed an initial decline at 6 months and a subsequent increase at 12 months follow‐up. However, BMD decreased in later follow‐up periods (24–60 months).

Data obtained from articles that used three ROI to assess the acetabular BMD were reported in Supporting Information: Annex A—Table S9. Articles using more than three ROI were excluded from the analysis to reduce the heterogeneity of the data.

### Total knee arthroplasty

**Figure 5:**
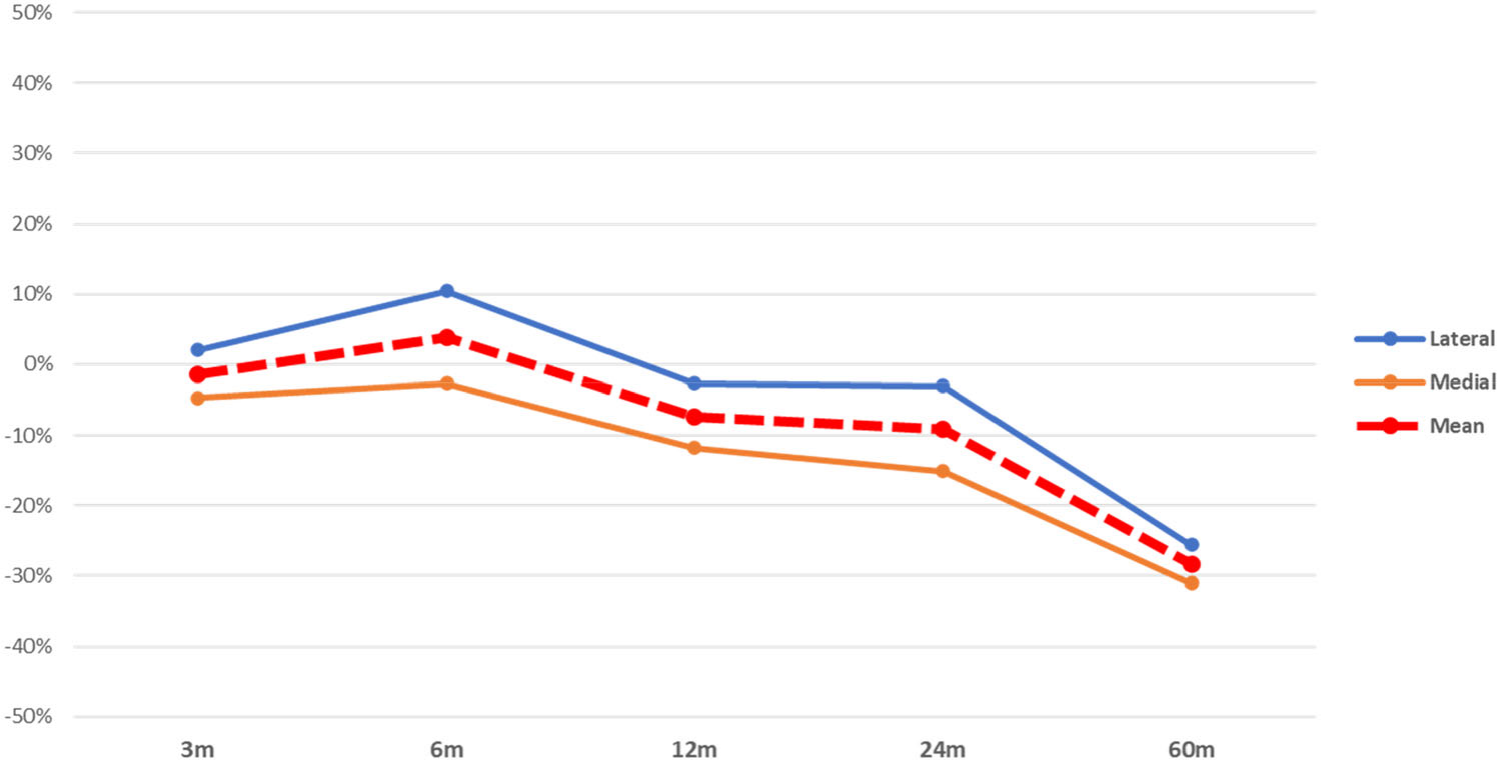
Overall BMD variation (%) after TKA. BMD, bone mineral density; TKA, total knee arthroplasty.

Studies analyzing the variation of BMD in knee replacements showed a high variability in the method of measurement. A systematic analysis of the data was only possible for studies concerning the BMD variation around the tibial component, where two ROIs, medial and lateral, were identified. Only data presented as g/cm^2^ were analysed, with a minimum sample of 50 knees. Finally, a total of 476 tibiae were included in this analysis, with an overall periprosthetic BMD (medial and lateral) of 0.95 g/cm^2^. On average, a steady decrease in BMD was observed around the tibial component at each follow‐up measurement (Table 5). However, the medial compartment showed a greater decrease than the lateral, which started to decrease after 12 months (Figure 5).

**Table 5:** Bone mineral density around tibial component after TKA.

ROI	Post surg	3 m		6 m		12 m		24 m		60 m	
	Mean (g/cm^2^)	Mean (g/cm^2^)	Difference (%)	Mean (g/cm^2^)	Difference (%)	Mean (g/cm^2^)	Difference (%)	Mean (g/cm^2^)	Difference (%)	Mean (g/cm^2^)	Difference (%)
Medial	0.95	0.91	−4.7%	0.93	−2.7%	0.84	−11.8%	0.81	−15.1%	0.66	−31.0%
Lateral	0.94	0.96	2.0%	1.04	10.4%	0.91	−2.7%	0.91	−3.0%	0.70	−25.6%
Mean	0.95	0.93	−1.4%	0.98	3.8%	0.88	−7.3%	0.86	−9.1%	0.68	−28.4%
Diff M‐L (%)	1.6%	−5.1%		−10.5%		−7.9%		−11.1%		−5.8%	
Knee (n°)	476	307		217		396		290		88	

Abbreviations: Diff, difference; L, lateral; M, medial; ROI, region of interest; TKA, total knee arthroplasty.

### Fixation technique in TKA

**Figure 6:**
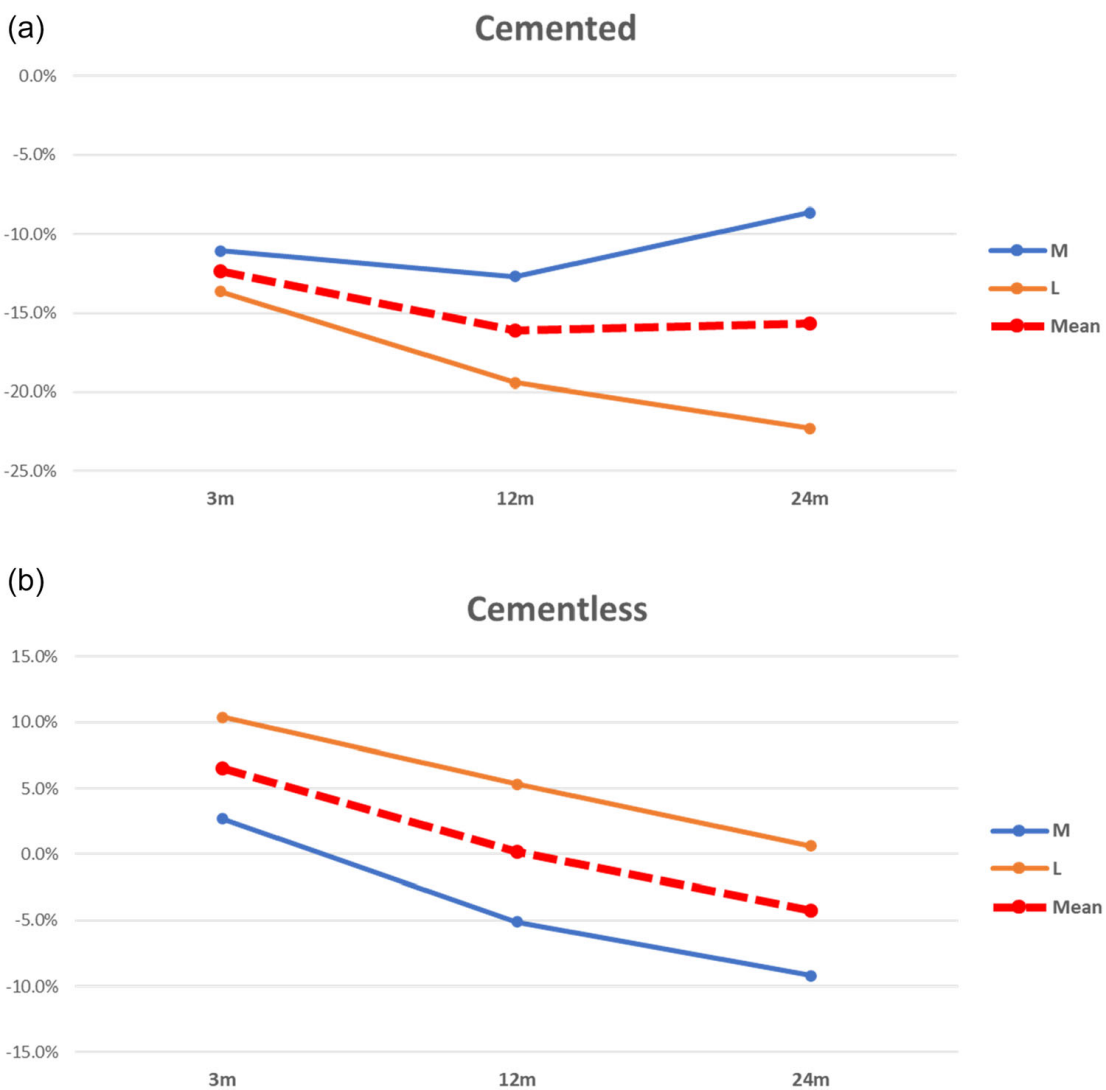
BMD comparison between cemented and cementless TKR across ROIs between the baseline and follow‐up times. *Note:* (a) Cemented knee prosthesis. (b) Cementless knee prosthesis. BMD, bone mineral density; ROI, region of interest; TKR, total knee replacement.

A total of 207 cemented and 269 cementless implants were examined, with 3, 12 and 24 months follow‐up (Table 6). Due to the small sample size, 6 months follow up was excluded from this analysis. Cemented implants showed a greater decrease in mean BMD at tibial level in each follow‐up than cementless implants. Furthermore, the greatest decrease in BMD was reported in the lateral compartment in cemented implants and in the medial compartment in cementless implants (Figure 6).

**Table 6:** BMD of tibial component after cemented versus cementless TKA.

	Cemented			Cementless		
ROI	3 m	12 m	24 m	3 m	12 m	24 m
M	−11.0%	−12.7%	−8.6%	2.7%	−5.1%	−9.2%
L	−13.6%	−19.4%	−22.3%	10.4%	5.3%	0.6%
Mean	−12.4%	−16.1%	−15.6%	6.6%	0.1%	−4.3%
Knee (n°)	106	168	62	201	228	228

Abbreviations: BMD, bone mineral density; ROI, region of interest; TKA, total knee arthroplasty.

### Implant design in TKA

**Figure 7:**
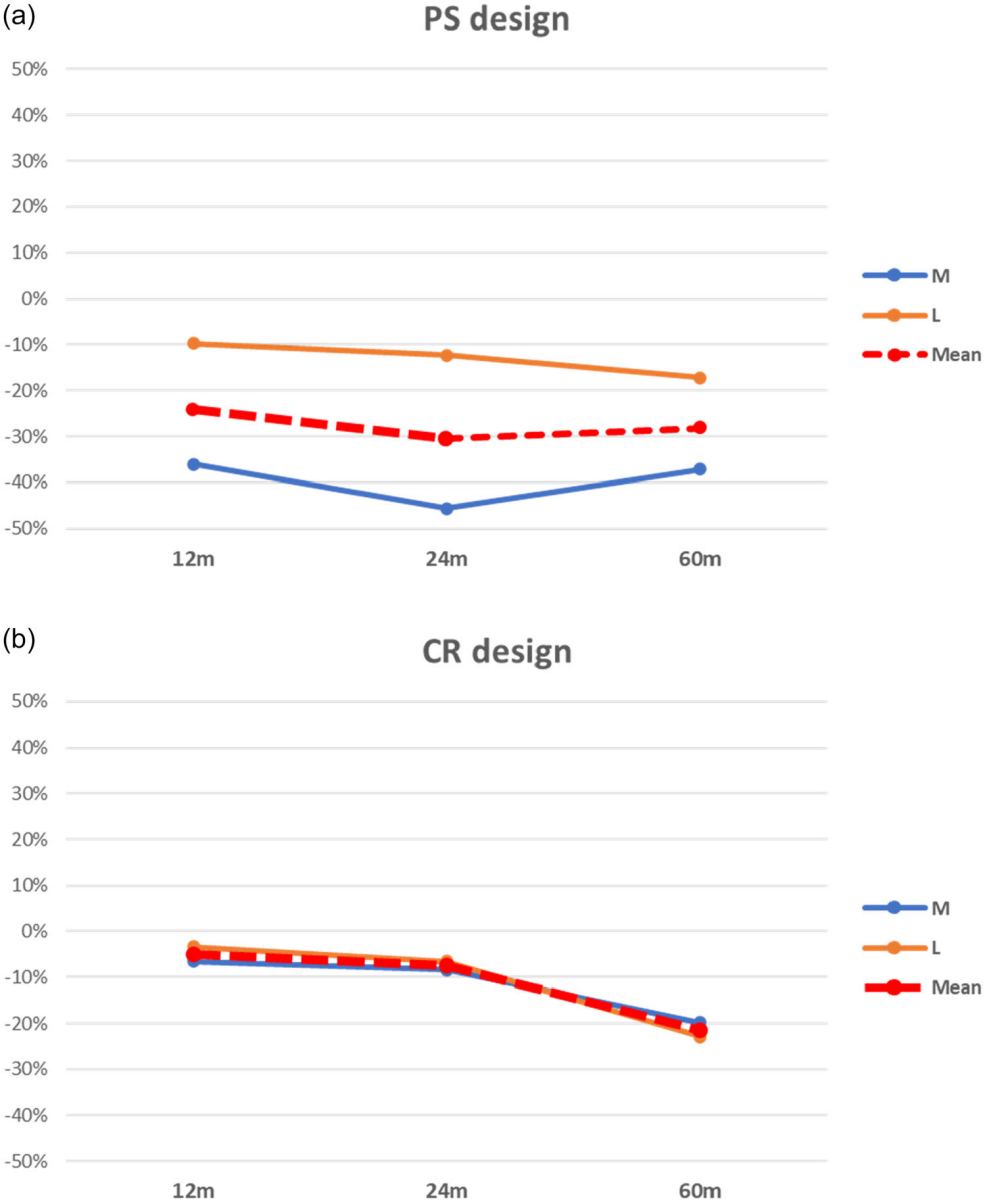
BMD Comparison between knee prosthesis design across ROIs between the baseline and follow‐up times. *Note:* (a) Posterior stabilised design. (b) Cruciate retaining design. BMD, bone mineral density; CR, cruciate retaining; PS, posterior stabilised; ROI, region of interest.

Data about BMD changes in posterior stabilised (PS) and cruciate retaining (CR) were analysed and compared. At baseline (post‐surgery), periprosthetic BMD was measured in 114 PS and 240 CR implants, then at 12, 24 and 60 months follow up (Table 7). PS implants showed a greater decrease in BMD than CR implants, with greater bone resorption in the medial compartment. On the other hand, CR implants showed a similar trend of BMD decrease between the two compartments (Figure 7).

**Table 7:** BMD of different knee prosthesis designs.

	PS			CR		
ROI	12 m	24 m	60 m	12 m	24 m	60 m
M	−36%	−10%	8%	−7%	−8%	−20%
L	−10%	−3%	−5%	−3%	−7%	−23%
Mean	−24%	−6%	2%	−5%	−7%	−21%
Knee (n°)	34	34	49	240	240	39

Abrreviations: BMD, bone mineral density; CR, cruciate retaining; PS, posterior stabilised; ROI, region of interest.

### Femoral component in TKA

Due to the absence of a standardised method for analyzing the variation of BMD in the femoral component, it was not feasible to conduct a comparative assessment of the results across different studies. However, the analysis of femoral BMD changes after TKA indicated greater overall bone resorption in the anterior femur, a region often susceptible to periprosthetic fractures, while one study comparing different types of inserts, showed more pronounced bone resorption at the level of posterior femoral condyles when using mobile bearing insert [65].

### Total shoulder arthroplasty

Only one study analysed periprosthetic bone in 22 shoulder arthroplasties [94]. The BMD was assessed at the humeral level, parallel to a line passing through the apex of the resurfacing implant. The BMD decreased by 22.4% from the baseline to 3 month‐follow up and of 1.4% to 6 month‐follow up (Supporting Information: Annex A ‐ Table S10).

## DISCUSSION

The main finding of this systematic review was that, after joint replacement, BMD changes depending on the anatomical region, fixation technique, and implant design. In THA, a significant overall bone resorption was reported at the level of the proximal femur. The use of cemented stems generally induced greater bone loss than cementless stems, with a rapid decrease in the first post‐operative months, then stabilized at mid‐term follow‐up. Anatomical stems better preserved BMD but with a higher risk of fractures and a more pronounced bone loss in the proximal femur compared with cemented stems.

In TKA, the medial tibial compartment and the anterior region of the distal femur reported the greatest BMD loss, while considering the fixation technique, cementless implants showed a lower bone loss compared to cemented implants. Additionally, posterior‐stabilised design produced a more pronounced bone resorption compared to cruciate‐retaining design.

### THA

The variation in BMD after THA was well documented and the use of Gruen zones allowed a direct comparison between different studies.

Regardless of the type of stem or fixation technique used, a negative peak of average BMD was reported 6 months after surgery, due to the adaptive response of bone to surgical stress [2, 9]. Analyzing Gruen's zones separately, it emerged how different patterns of load transfer produced a great bone resorption in the proximal femoral metaphysis (ROIs 1 and 7), a region subjected to a high strain energy density, while the Gruen zones 3, 4 and 5, showed a decrease in BMD at 6 months and an increase at 60 months compared to the baseline [38].

As demonstrated by Xu et al. [108] in a finite element analysis, the bone mass of the proximal femur presents a triangular high‐modulus distribution, which bears the main stress of the proximal femur. Our findings indicate that implanting a prosthesis with greater stiffness than bone shields the latter from absorbing loads, leading to stress shielding and a gradual depletion of the bone mineral matrix [108]. Furthermore, as discussed below, this phenomenon is also influenced by the fixation technique and implant design used.

### Fixation technique in THA

The use of cemented implants induced more bone resorption than cementless implants, with a marked difference at 6 months follow‐up. This phenomenon could be attributable to the thermic stress to the endosteal bone induced by cement polymerisation. However, the interface area of a cemented stem has been described as approximately 65 times greater than an uncemented calcar bearing stems [103, 104]. The uniform distribution of forces assured by the cement mantle could explain the preservation of BMD at the proximal femur in the medium term compared to cementless stems. A recent metanalysis comparing cemented and cementless THA did not demonstrate overall superiority of either method of fixation as measured by a difference in survival. However, it was found that cementless stems showed a higher survival rate in studies after 1995, while cemented stems showed a higher survival rate when considering studies not restricted to patients aged 55 or less [75]. This suggests that cemented stems should be preferred in elderly patients with poor bone quality that does not allow for proper osseointegration or that exposes them to the risk of intraoperative fractures, while modern uncemented stems should be implanted in younger patients in order to preserve the bone stock for the subsequent implant revision.

### Implant design in THA

Regarding the stem design, anatomical stems showed an overall better preservation of BMD than tapered stems, with a more pronounced BMD loss at the proximal femur at medium‐term follow‐up [54, 56]. This could be due to the stronger fixation on metaphyseal region of the anatomical stems compared to the wider and more distal distribution of the forces with tapered stems. Moreover, while the use of anatomical stems has increased in recent years driven by the advent of minimally invasive surgery and supported by the evidence of the preservation of bone stock and reduction of stress shielding [16, 52], an increased risk of periprosthetic fractures has also been reported [7, 27]. Hence, based on our results, anatomical stems should be preferred in young subjects with good bone quality. However, considering the described complications, careful consideration must be given to the quality of the recipient bone and to the implant sizing to prevent inadequate primary stability in osteoporotic patients or when implanting undersized stems, and post‐operative pain or intra‐operative fractures using oversized stems.

### Acetabular component

Only a few studies analysed BMD changes around the acetabular component. Such phenomenon is influenced by several factors like the type of implant and the specific regions of interest examined. It appears that initial declines in BMD are not uncommon but may stabilise or even reverse in certain regions over time, according to Wolff's law [106] and particularly with specific implant types (more pronounced BMD losses with threaded cups). Further research is likely needed to better understand the underlying mechanisms and clinical implications of these observed patterns.

### TKA

Data on BMD changes after TKA were more heterogeneous and a direct comparison between the various studies was only partially possible. Most of articles were focused on the tibia which, due to its geometry, is subject to higher peak forces and thus a higher rate of loosening than the femoral component, particularly in case of malalignment [42, 86]. In the studies analysed, the medial tibial compartment showed a higher decrease in BMD compared to lateral compartment in each follow up. From a kinematic point of view, the medial tibiofemoral compartment is exposed to higher contact force in the native knee [50, 51]. As described by Winther et al. [105], this leads to a greater BMD decrease in the medial tibia after TKA. A gap in the literature emerges from these findings that would be interesting to investigate. Can tibial component alignment influence BMD variation at the implant/bone interface? This would provide interesting insights into the safety of current kinematic/personalised alignments.

### Fixation technique in TKA

Analyzing fixation technique, cementless tibial components better preserved the BMD with respect to cemented implants, where the cementation technique, cement viscosity and other factors could influence the postoperative bone remodelling [85]. Furthermore, it was found that cemented implants showed a greater loss of BMD on the lateral compartment, whereas cementless tibial components showed a progressive BMD decrease in both medial and lateral compartments (Figure 6). However, the data available was not sufficient to generalise this behaviour, and further investigation is needed in future studies to explore this aspect thoroughly. Given the more extensive experience with cemented implants compared to cementless ones, cemented implants maintain their status as the gold standard in knee prosthetics.

### Implant design in TKA

Comparing the trend of the PS and CR designs, the former showed a greater decrease in BMD than CR implants, with greater bone resorption in the medial compartment. Kinematic studies showed that PS implants generate a more pronounced medial pivot in loaded knee flexion than CR implants, where the translation has been shown to be similar between the two compartments [11]. This could cause a different distribution of forces to the periprosthetic bone [107].

However, conventional symmetrical CR implants are more challenging to balance due to the variable tension of the posterior cruciate ligament, which can lead to instability through what is known as paradoxical anterior translation of the femur. In contrast, PS implants offer greater intrinsic stability, and their balancing is more reproducible. Furthermore, implants with a CR femoral component and ultra‐congruent or medially stabilised insert have been increasingly used in recent years, as they offer intrinsic stability comparable to PS implants. This could ensure greater preservation of periprosthetic BMD and will be investigated in future research by this study group.

### Femoral component in TKA

Analysis of femoral BMD changes after TKA showed increased bone resorption in the anterior portion of the femur, an area frequently subject to periprosthetic fractures [59, 99]. Moreover, it seems that mobile bearing TKA may better preserve BMD at the femoral level compared to fixed bearings. However, there is no strong evidence, and further investigation with a larger sample size is needed. Furthermore, studies involving SPECT for the evaluation of bone metabolism showed prolonged uptake at the level of the distal femur compared to the proximal tibia. This technique has been recently used to evaluate unhappy patients with pain, stiffness or swelling after TKA, showing potential for identifying typical patterns of bone tracer uptake for specific pathologies [40]. The use of SPECT in combination with DXA could be promising for investigating the influence of materials with lower stiffness on periprosthetic BMD at the femoral level.

### Total shoulder arthroplasty

The BMD trend after total shoulder arthroplasty was similar to that observed in other joints examined. However, the literature lacks comparisons of different designs and fixation techniques. This area deserves further investigation in future studies.

### Limitations

This systematic review has several limitations. BMD values at baseline were highly variable between different studies, and this may depend on the patient‐related factors (age, sex, comorbidities, pharmacological treatment, rehabilitation and level of physical activity), the quality of the bone tissue and the time between surgery and the first baseline DXA. These variables were not taken into account. Thus, to compare BMD trends between different studies, the percentage variation was considered for analysis instead of the nominal values. Moreover, this review has limitations arising from necessary deviations from the protocol to address methodological challenges and ensure the robustness of the review. These include excluding studies with non‐standard ROI methodologies or incomplete numerical data and applying minimum sample size thresholds to ensure robust analyses. While these adjustments reduced heterogeneity and improved data quality, they may have introduced a selection bias.

## CONCLUSION

This systematic review showed that periprosthetic BMD tends to decrease progressively after joint replacement surgery. The extent and pattern of this decline are influenced by the fixation technique and the implant design. These factors must be considered during the surgical planning, as they can have long‐term implications for bone health and implant longevity. Further research is necessary to optimise implant design and surgical techniques to mitigate BMD loss and improve patient outcomes.

## AUTHOR CONTRIBUTIONS

Laura Bragonzoni and Stefano Zaffagnini conceived and designed the study, developing the question guide. Raffaele Zinno and Giuseppe Barone formulated the search query. Domenico Alesi, Raffaele Zinno, Maria Scoppolini Massini, Giuseppe Barone, Davide Valente, Erika Pinelli, and Agostino Igor Mirulla contributed to abstract and full‐text screening. Maria Scoppolini Massini, Erika Pinelli, and Laura Bragonzoni conducted the quality assessment of the included studies. Domenico Alesi and Raffaele Zinno wrote the manuscript with contributions and critical revisions from all authors.

## CONFLICT OF INTEREST STATEMENT

The authors declare no conflicts of interest.

## ETHICS STATEMENT

None declared.

## Supporting information

Supporting information.

## Data Availability

The data supporting the findings of this study are derived from publicly available sources, including published articles and databases cited within this manuscript.
