# Supplementary material for: Variations in bone mineral density after joint replacement: A systematic review examining different anatomical regions, fixation techniques and implant design
Source: J Exp Orthop. 2025 May 20;12(2):e70187. doi: 10.1002/jeo2.70187 (PMC12092379; doi:10.1002/jeo2.70187)
Supplement: Supplementary file 1 — Supporting information. [file JEO2-12-e70187-s001.docx]

Annex A

Table S1. Search strings for Pubmed, Scopus, Cochrane, Web of Science, and Cinahl

| **Database** | **Search string** |
| --- | --- |
| Pubmed  n° 2,047  Filters:  English, Human | ("Bone mass"[Title/Abstract] OR "Bone turnover" [Title/Abstract] OR "Bone metabolism" [Title/Abstract] OR "Bone density"[Title/Abstract] OR "bone loss"[Title/Abstract] OR "bone losses"[Title/Abstract] OR "Bone Densities"[Title/Abstract] OR "Bone Mineral Density"[Title/Abstract] OR "Bone Mineral Densities"[Title/Abstract] OR "Bone Mineral Content"[Title/Abstract] OR "Bone Mineral Contents"[Title/Abstract] OR "BMD"[Title/Abstract]) AND ("arthroplasty, replacement, knee"[MeSH Terms] OR "Arthroplasty, Replacement, Ankle"[MeSH Terms] OR "Arthroplasty, Replacement, Elbow"[MeSH Terms] OR "Arthroplasty, Replacement, Shoulder"[MeSH Terms] OR "Arthroplasty, Replacement, Hip"[MeSH Terms]) |
| Scopus  n° 3,877  Filters:  **Inclusion:**  English  Article | (TITLE-ABS-KEY ("Bone mass") OR TITLE-ABS-KEY (“Bone turnover") OR TITLE-ABS-KEY ("Bone metabolism") OR TITLE-ABS-KEY (“Bone density") OR TITLE-ABS-KEY ("bone loss") OR TITLE-ABS-KEY ("bone losses") OR TITLE-ABS-KEY ("Bone Densities") OR TITLE-ABS-KEY ("Bone Mineral Density") OR TITLE-ABS-KEY ("Bone Mineral Densities") OR TITLE-ABS-KEY ("Bone Mineral Content") OR TITLE-ABS-KEY ("Bone Mineral Contents") OR TITLE-ABS-KEY (BMD)) AND (TITLE-ABS-KEY ("Hip Arthroplasty") OR TITLE-ABS-KEY ("Hip Prosthesis") OR TITLE-ABS-KEY ("Hip Prosthesis Implantation") OR TITLE-ABS-KEY ("Hip Prosthesis Implantations") OR TITLE-ABS-KEY ("Hip Replacement") OR TITLE-ABS-KEY ("Hip Replacement Arthroplasties") OR TITLE-ABS-KEY ("Hip Replacement Arthroplasty") OR TITLE-ABS-KEY ("Total Hip Arthroplasties") OR TITLE-ABS-KEY ("Total Hip Arthroplasty") OR TITLE-ABS-KEY ("Total Hip Replacement") OR TITLE-ABS-KEY ("Total Hip Replacements") ORTITLE-ABS-KEY ("Ankle Replacement Arthroplasty") OR TITLE-ABS-KEY ("Ankle Replacement Arthroplasties") OR TITLE-ABS-KEY ("Ankle Replacement") OR TITLE-ABS-KEY ("Total Ankle Replacement") OR TITLE-ABS-KEY ("Ankle Replacements") OR TITLE-ABS-KEY ("Total Ankle Replacements") OR TITLE-ABS-KEY ("Knee Replacement") OR TITLE-ABS-KEY ("Knee Replacement Arthroplasties") OR TITLE-ABS-KEY ("Knee Replacement Arthroplasty") OR TITLE-ABS-KEY ("Replacement Arthroplasties") OR TITLE-ABS-KEY ("Knee Arthroplasty") OR TITLE-ABS-KEY ("Total Knee Arthroplasty") OR TITLE-ABS-KEY ("Total Knee Replacement") OR TITLE-ABS-KEY ("Unicompartmental Knee") OR TITLE-ABS-KEY ("Replacement Arthroplasty") OR TITLE-ABS-KEY ("Unicompartmental Knee Arthroplasty") OR TITLE-ABS-KEY ("Unicondylar Knee Arthroplasty") OR TITLE-ABS-KEY ("Unicondylar Knee") OR TITLE-ABS-KEY ("Partial Knee Arthroplasty") OR TITLE-ABS-KEY ("Unicondylar Knee Replacement") OR TITLE-ABS-KEY ("Partial Knee Replacement") OR TITLE-ABS-KEY ("Unicompartmental Knee Replacement") OR TITLE-ABS-KEY ("Total Shoulder Replacement") OR TITLE-ABS-KEY ("Total Shoulder") OR TITLE-ABS-KEY ("Shoulder Replacement Arthroplasties") OR TITLE-ABS-KEY ("Shoulder Replacement") OR TITLE-ABS-KEY ("Shoulder Replacements") OR TITLE-ABS-KEY ("Total Shoulder Replacements") OR TITLE-ABS-KEY ("Shoulder Replacement Arthroplasty") OR TITLE-ABS-KEY ("Elbow Replacement Arthroplasty") OR TITLE-ABS-KEY ("Elbow Replacement") OR TITLE-ABS-KEY ("Elbow Replacement Arthroplasties") OR TITLE-ABS-KEY ("Total Elbow Replacements") OR TITLE-ABS-KEY ("Total Elbow Replacement") OR TITLE-ABS-KEY ("Elbow Replacements") |
| Cochrane  n° 442  Filters:  **Exclusion:**  Review  protocols | (("Bone mass" OR "Bone turnover" OR "Bone metabolism" OR "Bone density" OR "bone loss" OR "bone losses" OR "Bone Densities" OR "Bone Mineral Density" OR "Bone Mineral Densities" OR "Bone Mineral Content" OR "Bone Mineral Contents" OR "BMD") AND ("Hip Arthroplasty" OR "Hip Prosthesis" OR "Hip Prosthesis Implantation" OR "Hip Prosthesis Implantations" OR "Hip Replacement" OR "Hip Replacement Arthroplasties" OR "Hip Replacement Arthroplasty" OR "Total Hip Arthroplasties" OR "Total Hip Arthroplasty" OR "Total Hip Replacement" OR "Total Hip Replacements" OR "Ankle Replacement Arthroplasty" OR "Ankle Replacement Arthroplasties" OR "Ankle Replacement" OR "Total Ankle Replacement" OR "Ankle Replacements" OR "Total Ankle Replacements" OR "Knee Replacement" OR "Knee Replacement Arthroplasties" OR "Knee Replacement Arthroplasty" OR "Replacement Arthroplasties" OR "Knee Arthroplasty" OR "Total Knee Arthroplasty" OR "Total Knee Replacement" OR "Unicompartmental Knee" OR "Replacement Arthroplasty" OR "Unicompartmental Knee Arthroplasty" OR "Unicondylar Knee Arthroplasty" OR "Unicondylar Knee" OR "Partial Knee Arthroplasty" OR "Unicondylar Knee Replacement" OR "Partial Knee Replacement" OR "Unicompartmental Knee Replacement" OR "Total Shoulder Replacement" OR "Total Shoulder" OR "Shoulder Replacement Arthroplasties" OR "Shoulder Replacement" OR "Shoulder Replacements" OR "Total Shoulder Replacements" OR "Shoulder Replacement Arthroplasty" OR "Elbow Replacement Arthroplasty" OR "Elbow Replacement" OR "Elbow Replacement Arthroplasties" OR "Total Elbow Replacements" OR "Total Elbow Replacement" OR "Elbow Replacements")):ti,ab,kw |
| Web of Science  n° 1917  Filters:  **Inclusion:**  English  **Exclusion:**  Review  Book  Conference | ((((((((((((TI=("Bone mass")) OR TI=("Bone turnover")) OR TI=("Bone metabolism")) OR TI=("Bone density")) OR TI=("bone loss")) OR TI=("bone losses")) OR TI=("Bone Densities")) OR TI=("Bone Mineral Density")) OR TI=("Bone Mineral Densities")) OR TI=("Bone Mineral Content")) OR TI=("Bone Mineral Contents")) OR TI=("BMD")) AND ((((((((((((((((((((((((((((((((((((((((((((((TI=("Hip Arthroplasty")) OR TI=("Hip Prosthesis")) OR TI=("Hip Prosthesis Implantation")) OR TI=("Hip Prosthesis Implantations")) OR TI=("Hip Replacement")) OR TI=("Hip Replacement Arthroplasties")) OR TI=("Hip Replacement Arthroplasty")) OR TI=("Total Hip Arthroplasties")) OR TI=("Total Hip Arthroplasty")) OR TI=("Total Hip Replacement")) OR TI=("Total Hip Replacements")) OR TI=("Ankle Replacement Arthroplasty")) OR TI=("Ankle Replacement Arthroplasties")) OR TI=("Ankle Replacement")) OR TI=("Total Ankle Replacement")) OR TI=("Ankle Replacements")) OR TI=("Total Ankle Replacements")) OR TI=("Knee Replacement")) OR TI=("Knee Replacement Arthroplasties")) OR TI=("Knee Replacement Arthroplasty")) OR TI=("Replacement Arthroplasties")) OR TI=("Knee Arthroplasty")) OR TI=("Total Knee Arthroplasty")) OR TI=("Total Knee Replacement")) OR TI=("Unicompartmental Knee")) OR TI=("Replacement Arthroplasty")) OR TI=("Unicompartmental Knee Arthroplasty")) OR TI=("Unicondylar Knee Arthroplasty")) OR TI=("Unicondylar Knee")) OR TI=("Partial Knee Arthroplasty")) OR TI=("Unicondylar Knee Replacement")) OR TI=("Partial Knee Replacement")) OR TI=("Unicompartmental Knee Replacement")) OR TI=("Total Shoulder Replacement")) OR TI=("Total Shoulder")) OR TI=("Shoulder Replacement Arthroplasties")) OR TI=("Shoulder Replacement")) OR TI=("Shoulder Replacements")) OR TI=("Total Shoulder Replacements")) OR TI=("Shoulder Replacement Arthroplasty")) OR TI=("Elbow Replacement Arthroplasty")) OR TI=("Elbow Replacement")) OR TI=("Elbow Replacement Arthroplasties")) OR TI=("Total Elbow Replacements")) OR TI=("Total Elbow Replacement")) OR TI=("Elbow Replacements"))  ((((((((((((AB=("Bone mass")) OR AB=("Bone turnover")) OR AB=("Bone metabolism")) OR AB=("Bone density")) OR AB=("bone loss")) OR AB=("bone losses")) OR AB=("Bone Densities")) OR AB=("Bone Mineral Density")) OR AB=("Bone Mineral Densities")) OR AB=("Bone Mineral Content")) OR AB=("Bone Mineral Contents")) OR AB=("BMD")) AND ((((((((((((AB=("Bone mass")) OR AB=("Bone turnover")) OR AB=("Bone metabolism")) OR AB=("Bone density")) OR AB=("bone loss")) OR AB=("bone losses")) OR AB=("Bone Densities")) OR AB=("Bone Mineral Density")) OR AB=("Bone Mineral Densities")) OR AB=("Bone Mineral Content")) OR AB=("Bone Mineral Contents")) OR AB=("BMD")) AND ((((((((((((((((((((((((((((((((((((((((((((((AB=(“Hip Arthroplasty")) OR AB=("Hip Prosthesis")) OR AB=("Hip Prosthesis Implantation")) OR AB=("Hip Prosthesis Implantations")) OR AB=("Hip Replacement")) OR AB=("Hip Replacement Arthroplasties")) OR AB=("Hip Replacement Arthroplasty")) OR AB=("Total Hip Arthroplasties")) OR AB=("Total Hip Arthroplasty")) OR AB=("Total Hip Replacement")) OR AB=("Total Hip Replacements")) OR AB=("Ankle Replacement Arthroplasty")) OR AB=("Ankle Replacement Arthroplasties")) OR AB=("Ankle Replacement")) OR AB=("Total Ankle Replacement")) OR AB=("Ankle Replacements")) OR AB=("Total Ankle Replacements")) OR AB=("Knee Replacement")) OR AB=("Knee Replacement Arthroplasties")) OR AB=("Knee Replacement Arthroplasty")) OR AB=("Replacement Arthroplasties")) OR AB=("Knee Arthroplasty")) OR AB=("Total Knee Arthroplasty")) OR AB=("Total Knee Replacement")) OR AB=("Unicompartmental Knee")) OR AB=("Replacement Arthroplasty")) OR AB=("Unicompartmental Knee Arthroplasty")) OR AB=("Unicondylar Knee Arthroplasty")) OR AB=("Unicondylar Knee")) OR AB=("Partial Knee Arthroplasty")) OR AB=("Unicondylar Knee Replacement")) OR AB=("Partial Knee Replacement")) OR AB=("Unicompartmental Knee Replacement")) OR AB=("Total Shoulder Replacement")) OR AB=("Total Shoulder")) OR AB=("Shoulder Replacement Arthroplasties")) OR AB=("Shoulder Replacement")) OR AB=("Shoulder Replacements")) OR AB=("Total Shoulder Replacements")) OR AB=("Shoulder Replacement Arthroplasty")) OR AB=("Elbow Replacement Arthroplasty")) OR AB=("Elbow Replacement")) OR AB=("Elbow Replacement Arthroplasties")) OR AB=("Total Elbow Replacements")) OR AB=("Total Elbow Replacement")) OR AB=("Elbow Replacements")) |
| Cinahl  n° 875  Filters:  English  Human | (Bone mass OR Bone turnover OR Bone metabolism OR Bone density OR bone loss OR bone losses OR Bone Densities OR Bone Mineral Density OR Bone Mineral Densities OR Bone Mineral Content OR Bone Mineral Contents OR BMD) AND (Hip Arthroplasty OR Hip Prosthesis OR Hip Prosthesis Implantation OR Hip Prosthesis Implantations OR Hip Replacement OR Hip Replacement Arthroplasties OR Hip Replacement Arthroplasty OR Total Hip Arthroplasties OR Total Hip Arthroplasty OR Total Hip Replacement OR Total Hip Replacements OR Ankle Replacement Arthroplasty OR Ankle Replacement Arthroplasties OR Ankle Replacement OR Total Ankle Replacement OR Ankle Replacements OR Total Ankle Replacements OR Knee Replacement OR Knee Replacement Arthroplasties OR Knee Replacement Arthroplasty OR Replacement Arthroplasties OR Knee Arthroplasty OR Total Knee Arthroplasty OR Total Knee Replacement OR Unicompartmental Knee OR Replacement Arthroplasty OR Unicompartmental Knee Arthroplasty OR Unicondylar Knee Arthroplasty OR Unicondylar Knee OR Partial Knee Arthroplasty OR Unicondylar Knee Replacement OR Partial Knee Replacement OR Unicompartmental Knee Replacement OR Total Shoulder Replacement OR Total Shoulder OR Shoulder Replacement Arthroplasties OR Shoulder Replacement OR Shoulder Replacements OR Total Shoulder Replacements OR Shoulder Replacement Arthroplasty OR Elbow Replacement Arthroplasty OR Elbow Replacement OR Elbow Replacement Arthroplasties OR Total Elbow Replacements OR Total Elbow Replacement OR Elbow Replacements) |

Table S2. Joanna Briggs Institute (JBI) critical appraisal checklist for analytical cross-sectional studies

| References | 1. Were the criteria for inclusion in the sample clearly defined? | 2. Were the study subjects and the setting described in detail? | 3. Was the exposure measured in a valid and reliable way? | 4. Were objective, standard criteria used for measurement of the condition? | 5. Were confounding factors identified? | 6. Were strategies to deal with confounding factors stated? | 7. Were the outcomes measured in a valid and reliable way? | 8. Was appropriate statistical analysis used? |
| --- | --- | --- | --- | --- | --- | --- | --- | --- |
| Aldinger, P. R., 2003 | Y | Y | Y | Y | N | N | Y | Y |
| Alm, J., 2009 | Y | Y | Y | Y | Y | Y | Y | Y |
| Andersen, M., 2018 | N | Y | Y | Y | N | N | Y | Y |
| Brinkmann, V. 2017 | Y | Y | Y | Y | N | N | Y | Y |
| Burchard, R., 2007 | Y | Y | Y | Y | N | N | Y | U |
| Christiansen, J., 2020 | Y | Y | Y | Y | Y | Y | Y | Y |
| Damborg, F., 2008 | Y | Y | Y | Y | N | N | Y | Y |
| Decking, R., 2008 | N | Y | Y | Y | N | N | Y | Y |
| Digas, G et al. 2009 | N | Y | Y | Y | Y | Y | Y | Y |
| Ebert, J et al. 2022 | Y | Y | U | Y | N | N | Y | Y |
| Field, R et al. 2006 | Y | N | Y | Y | N | N | Y | U |
| Fischer, M et al. 2017 | Y | Y | Y | Y | N | N | Y | Y |
| Gazdzik, T et al. 2008 | Y | Y | Y | Y | N | N | N | U |
| Herrera Et Al. 2014 | Y | Y | Y | Y | N | N | Y | Y |
| Jahnke 2014 | Y | Y | Y | Y | Y | Y | Y | Y |
| Kim, Y et al. 2014 | Y | Y | Y | U | N | N | Y | Y |
| Leichtle, U et al. 2006 | Y | Y | Y | Y | Y | N | Y | Y |
| Lerch, M et al. 2012 a | Y | Y | Y | Y | N | N | Y | Y |
| Lerch, M et al. 2012 b | Y | U | Y | Y | N | N | Y | Y |
| Lerch, M et al. 2012 c | Y | Y | Y | Y | N | N | Y | Y |
| Minoda, Y et al. 2022 b | N | Y | U | N | Y | Y | Y | Y |
| Morita, D et al. 2016 | N | Y | Y | Y | Y | Y | Y | Y |
| Nyström, A et al. 2022 | Y | Y | Y | Y | N | N | Y | Y |
| Pandit, S et al, 2006 | U | Y | Y | Y | N | N | Y | U |
| Pitto, R et al, 2008 | Y | U | Y | Y | N | N | Y | Y |
| Pitto, R et al, 2010 | Y | Y | Y | Y | Y | U | Y | Y |
| Synder, M et al. 2015 | Y | Y | Y | Y | N | N | Y | Y |
| Soininvaara, T et al. 2008 | U | Y | Y | Y | N | N | U | Y |
| Soininvaara, T et al. 2013 | Y | Y | Y | Y | N | N | Y | Y |
| Steens, W et al. 2015 | U | Y | Y | Y | N | N | Y | Y |
| Tapaninen 2012 | Y | Y | Y | Y | N | N | U | Y |
| Venesmaa, P et al. 2003 | Y | Y | Y | Y | Y | U | Y | Y |

Note: Y: Yes; N: No; U: Unclear; NA: Not applicable.

Table S3. Joanna Briggs Institute (JBI) critical appraisal checklist for cohort studies

| References | 1. Were the two groups similar and recruited from the same population? | 2. Were the exposures measured similarly to assign people to both exposed and unexposed groups? | 3. Was the exposure measured in a valid and reliable way? | 4. Were confounding factors identified? | 5. Were strategies to deal with confounding factors stated? | 6. Were the groups/participants free of the outcome at the start of the study (or at the moment of exposure)? | 7. Were the outcomes measured in a valid and reliable way? | 8. Was the follow up time reported and sufficient to be long enough for outcomes to occur? | 9. Was follow up complete, and if not, were the reasons to loss to follow up described and explored? | 10. Were strategies to address incomplete follow up utilized? | 11. Was appropriate statistical analysis used? |
| --- | --- | --- | --- | --- | --- | --- | --- | --- | --- | --- | --- |
| Boller, S et al. 2018 | N | Y | Y | Y | N | N | Y | Y | N | N | Y |
| Digas, G et al. 2006 | Y | Y | Y | Y | N | Y | Y | Y | Y | N | Y |
| Galli, M et al. 2008 | U | Y | Y | N | N | U | Y | Y | N | N | Y |
| Grochola, L et al. 2008 | N | Y | Y | N | N | U | N | Y | N | N | Y |
| Hayaishi et al. 2007 | U | Y | Y | N | U | U | Y | Y | N | N | Y |
| Herrera et al. 2007 | U | Y | Y | N | N | Y | Y | Y | U | N | N |
| Huang et al. 2013 | Y | Y | Y | N | N | Y | Y | Y | U | N | Y |
| Koppens, D et al. 2020 | U | Y | Y | N | N | Y | Y | Y | N | N | Y |
| Liu, Y et al. 2022 | Y | Y | Y | N | N | Y | Y | Y | N | N | Y |
| López-Subías, J et al. 2019 | Y | Y | Y | N | N | Y | Y | Y | N | N | Y |
| Merle, C et al. 2012 | Y | Y | Y | Y | N | Y | Y | Y | Y | N | Y |
| Meyer, J Et Al. 2020 | N | Y | Y | N | N | Y | Y | Y | N | Y | Y |
| Minoda, Y et al. 2013 | Y | Y | U | Y | Y | U | Y | Y | N | N | Y |
| Minoda, Y et al. 2020 | Y | Y | U | N | N | U | Y | Y | N | N | Y |
| Minoda, Y et al. 2022 | Y | Y | Y | U | U | U | U | Y | N | N | Y |
| Panisello, J et al. 2009 | U | Y | Y | N | N | U | Y | Y | U | N | Y |
| Panisello, J et al. 2009 b | Y | Y | Y | Y | N | Y | Y | Y | Y | Y | Y |

Note: Y: Yes; N: No; U: Unclear; NA: Not applicable.

Table S4. Joanna Briggs Institute (JBI) critical appraisal checklist for case series

| Authors | 1. Were there clear criteria for inclusion in the case series? | 2. Was the condition measured in a standard, reliable way for all participants included in the case series? | 3. Were valid methods used for identification of the condition for all participants included in the case series? | 4. Did the case series have consecutive inclusion of participants? | 5. Did the case series have complete inclusion of participants? | 6. Was there clear reporting of the demographics of the participants in the study? | 7. Was there clear reporting of clinical information of the participants? | 8. Were the outcomes or follow up results of cases clearly reported? | 9. Was there clear reporting of the presenting site(s)/clinic(s) demographic information? | 10. Was statistical analysis appropriate? |
| --- | --- | --- | --- | --- | --- | --- | --- | --- | --- | --- |
| Buckland, A et al. 2010 | Y | Y | Y | Y | N | N | Y | N | Y | Y |

Note: Y: Yes; N: No; U: Unclear; NA: Not applicable.

Table S5. Joanna Briggs Institute (JBI) critical appraisal checklist for quasi-experimental studies

| References | 1. Is it clear in the study what is the ‘cause’ and what is the ‘effect’ (i.e. there is no confusion about which variable comes first)? | 2. Were the participants included in any comparisons similar? | 3. Were the participants included in any comparisons receiving similar treatment/care, other than the exposure or intervention of interest? | 4. Was there a control group? | 5. Were there multiple measurements of the outcome both pre and post the intervention/exposure? | 6. Was follow up complete and if not, were differences between groups in terms of their follow up adequately described and analyzed? | 7. Were the outcomes of participants included in any comparisons measured in the same way? | 8. Were outcomes measured in a reliable way? | 9. Was appropriate statistical analysis used? |
| --- | --- | --- | --- | --- | --- | --- | --- | --- | --- |
| Bieger, R et al. 2011 | Y | NA | N | N | Y | N | Y | Y | Y |
| Dan, D et al. 2006 | Y | N | N | N | Y | Y | Y | Y | Y |

Note: Y: Yes; N: No; U: Unclear; NA: Not applicable.

Table S6. Revised cochrane risk of bias tool for randomized trials (ROB 2)

| Authors | Risk Of Bias |
| --- | --- |
| Brinkmann, V et al. 2015 | High |
| Freitag, T et al. 2016 | Some Concerns |
| Gauthier, L et al. 2013 | Some Concerns |
| Kim, Y Et Al. 2007 | High |
| Kim, Y Et Al. 2011 | Some Concerns |
| Macdonald, S Et Al. 2010 | Some Concerns |
| Meyer, J Et Al. 2019 | Some Concerns |
| Motomura, G Et Al. 2022 | Some Concerns |
| Nysted, M Et Al. 2011 | Some Concerns |
| Rathsach Andersen, M Et Al. 2019 | High |
| Saari, T Et Al. 2007 | High |
| Stilling, M Et Al. 2012 | High |
| Ten Broeke, R Et Al. 2012 | Some Concerns |
| Vidovic, D Et Al. 2013 | Some Concerns |
| Winther, N Et Al. 2016 | Some Concerns |
| Zerahn, B Et Al. 2011 | Some Concerns |

Table S7. Temporal Variation in Bone Mineral Density across Regions of Interest during Follow-up Periods (g/cm²)

|  |  | **Post surg** | **1.5m** | **3m** | **6m** | **9m** | **12m** | **18m** | **24m** | **36m** | **42m** | **48m** | **54m** | **60m** | **72m** | **84m** | **96m** | **120m** | **156m** | **180m** |
| --- | --- | --- | --- | --- | --- | --- | --- | --- | --- | --- | --- | --- | --- | --- | --- | --- | --- | --- | --- | --- |
|  | **n°** | 3473 | 49 | 898 | 1383 | 103 | 2255 | 121 | 1277 | 477 | 43 | 265 | 43 | 836 | 61 | 35 | 21 | 122 | 35 | 500 |
| **ROI** | **1** | 0.90 | 0.94 | 0.83 | 0.72 | 1.09 | 0.78 | 1.05 | 0.83 | 0.74 | 0.76 | 1.17 | 0.75 | 0.81 | 0.61 | 0.71 | 0.82 | 0.59 | 0.69 | 0.99 |
|  | **2** | 1.55 | 1.66 | 1.48 | 1.42 | 1.67 | 1.51 | 1.64 | 1.55 | 1.38 | 1.53 | 1.93 | 1.51 | 1.52 | 1.18 | 1.30 | 1.19 | 1.13 | 1.30 | 1.08 |
|  | **3** | 1.74 | 1.66 | 1.83 | 1.67 | 1.91 | 1.82 | 1.86 | 1.79 | 1.69 | 1.86 | 2.09 | 1.86 | 1.85 | 1.42 | 1.56 | 2.15 | 1.35 | 1.60 | 1.06 |
|  | **4** | 1.75 | 1.77 | 1.87 | 1.71 | 1.98 | 1.84 | 1.97 | 1.84 | 1.69 | 1.72 | 2.30 | 1.74 | 1.84 | 1.46 | 1.64 | 1.94 | 1.43 | 1.60 | 1.04 |
|  | **5** | 1.79 | 1.72 | 1.84 | 1.72 | 1.95 | 1.85 | 1.90 | 1.83 | 1.68 | 1.94 | 1.99 | 1.98 | 1.87 | 1.48 | 1.71 | 2.14 | 1.44 | 1.67 | 1.04 |
|  | **6** | 1.51 | 1.61 | 1.48 | 1.41 | 1.74 | 1.48 | 1.66 | 1.51 | 1.32 | 1.57 | 2.48 | 1.56 | 1.51 | 1.33 | 1.34 | 1.19 | 1.32 | 1.33 | 0.96 |
|  | **7** | 1.21 | 1.10 | 1.06 | 1.00 | 1.18 | 1.05 | 1.19 | 1.05 | 0.94 | 1.04 | 1.29 | 1.00 | 1.09 | 0.73 | 0.84 | 0.94 | 0.67 | 0.80 | 0.88 |
|  | **Mean** | 1.49 | 1.50 | 1.48 | 1.38 | 1.65 | 1.48 | 1.61 | 1.49 | 1.35 | 1.49 | 1.89 | 1.49 | 1.50 | 1.17 | 1.30 | 1.48 | 1.14 | 1.29 | 1.01 |

Table S8. Percentage differences in Bone Mineral Density compared to the post surgery evaluation time point

|  |  | **1.5m** | **3m** | **6m** | **9m** | **12m** | **18m** | **24m** | **36m** | **42m** | **48m** | **54m** | **60m** | **72m** | **84m** | **96m** | **120m** | **156m** | **180m** |
| --- | --- | --- | --- | --- | --- | --- | --- | --- | --- | --- | --- | --- | --- | --- | --- | --- | --- | --- | --- |
| **ROI** | **1** | 4.7% | -7.8% | -19.3% | 21.7% | -12.7% | 17.1% | -7.7% | -17.5% | -15.2% | 30.2% | -16.3% | -9.7% | -32.2% | -20.9% | -8.5% | -33.7% | -23.3% | 10.6% |
|  | **2** | 13.1% | -7.7% | -14.1% | 13.8% | -4.6% | 10.4% | 0.0% | -18.8% | -1.9% | 43.2% | -4.1% | -2.5% | -40.9% | -27.8% | -39.8% | -46.1% | -27.1% | -52.0% |
|  | **3** | -9.2% | 9.8% | -8.5% | 18.7% | 8.8% | 13.3% | 5.3% | -6.2% | 13.1% | 38.5% | 13.1% | 12.0% | -36.0% | -20.2% | 45.5% | -43.6% | -15.6% | -76.1% |
|  | **4** | 2.4% | 13.8% | -4.1% | 25.9% | 10.1% | 24.6% | 10.5% | -6.1% | -3.1% | 61.4% | -0.9% | 10.4% | -32.2% | -11.9% | 21.4% | -35.2% | -16.3% | -78.7% |
|  | **5** | -7.4% | 6.4% | -7.0% | 18.3% | 6.8% | 12.8% | 5.4% | -11.4% | 17.2% | 22.7% | 21.6% | 9.4% | -34.2% | -8.9% | 39.5% | -38.5% | -12.7% | -83.2% |
|  | **6** | 11.3% | -3.8% | -12.1% | 25.3% | -3.6% | 16.5% | -0.8% | -21.4% | 6.3% | 108.2% | 5.2% | -0.7% | -20.5% | -19.1% | -36.1% | -21.2% | -20.6% | -61.5% |
|  | **7** | -11.9% | -16.5% | -24.0% | -3.5% | -18.0% | -2.1% | -17.8% | -30.5% | -19.1% | 8.8% | -23.6% | -13.8% | -53.4% | -41.0% | -30.3% | -60.5% | -46.0% | -36.7% |
|  | **Mean** | 0.3% | -0.5% | -7.6% | 10.3% | -1.1% | 7.9% | -0.4% | -9.6% | -0.2% | 26.9% | -0.4% | 0.4% | -21.4% | -12.9% | -0.7% | -23.9% | -13.9% | -32.4% |

Note: Negative value indicates a loss of BMD.

Table S9. BMD changes around acetabular component after THA

| ROI | Post surg | 6m |  | 12m |  | 24m |  | 60m |  |
| --- | --- | --- | --- | --- | --- | --- | --- | --- | --- |
|  | Mean (g/cm^2^) | Mean (g/cm^2^) | Difference (%) | Mean (g/cm^2^) | Difference (%) | Mean (g/cm^2^) | Difference (%) | Mean (g/cm^2^) | Difference (%) |
| 1 | 1.08 | 1.29 | 20.0% | 1.32 | 22.5% | 1.37 | 26.7% | 1.52 | 41.1% |
| 2 | 1.01 | 1.10 | 9.4% | 1.08 | 7.2% | 1.04 | 3.2% | 0.95 | -5.7% |
| 3 | 1.02 | 0.94 | -7.8% | 1.02 | 0.1% | 0.93 | -9.0% | 1.01 | -0.8% |
| Mean | 1.04 | 1.11 | 7.4% | 1.14 | 10.2% | 1.11 | 7.4% | 1.16 | 12.2% |
| Cup of hip (n°) | 609 | 59 |  | 59 |  | 59 |  | 50 |  |

Table S10. BMD of total shoulder arthroplasty

| ROI | Post surg | 3m |  | 6m |  |
| --- | --- | --- | --- | --- | --- |
|  | Mean (g/cm^2^) | Mean (g/cm^2^) | Difference (%) | Mean (g/cm^2^) | Difference (%) |
| 1 | 0.52 | 0.40 | -22.4% | 0.40 | -1.4% |
| Shoulder (n°) | 22 | 22 |  | 22 |  |
